# Nucleolar fibrillarin methyltransferase regulates systemic trafficking of a plant virus satellite RNA

**DOI:** 10.1093/plcell/koaf224

**Published:** 2025-09-22

**Authors:** Chih-Hao Chang, Jiun-Da Wang, Shu-Chuan Lee, Yau-Heiu Hsu, Chung-Chi Hu, Na-Sheng Lin

**Affiliations:** Institute of Plant and Microbial Biology, Academia Sinica, Taipei 11529, Taiwan, Republic of China; Institute of Plant and Microbial Biology, Academia Sinica, Taipei 11529, Taiwan, Republic of China; Institute of Plant and Microbial Biology, Academia Sinica, Taipei 11529, Taiwan, Republic of China; Graduate Institute of Biotechnology, National Chung Hsing University, Taichung 40227, Taiwan, Republic of China; Graduate Institute of Biotechnology, National Chung Hsing University, Taichung 40227, Taiwan, Republic of China; Institute of Plant and Microbial Biology, Academia Sinica, Taipei 11529, Taiwan, Republic of China; Department of Biotechnology and Bioindustry Sciences, Department of Life Sciences, National Cheng Kung University, Tainan 70101, Taiwan, Republic of China

## Abstract

RNA trafficking is crucial in almost every phase of plant development. Fibrillarin (FIB), a highly conserved nucleolar protein with methyltransferase (MTase) activity, functions in methylation and rRNA processing and facilitates the transport of several RNA viruses in plants. Previously, we demonstrated that bamboo mosaic virus satellite RNA (satBaMV) traffics autonomously and systemically in a helper virus-independent but FIB-dependent manner by forming a mobile ribonucleoprotein (RNP) complex comprising satBaMV, FIB, and satBaMV-encoded P20 movement protein. Here, we show that FIB methylates the arginine-rich motif (ARM) of P20 and relies on its MTase activity for the systemic movement of satBaMV. FIB MTase-defective mutants failed to complement long-distance satBaMV transport in FIB*i* plants, despite still binding to satBaMV in vivo. We also demonstrate that the ARM of P20 guides its nucleolar localization for FIB-mediated methylation. P20 methylation not only contributes to its plasmodesmata (PD) targeting but also triggers nucleocytoplasmic shuttling of FIB with P20 as the RNP complex to PD. A satBaMV mutant harboring a nonmethylated P20, but not a methylation-mimic P20, exhibited disrupted PD targeting and impaired P20-assisted satBaMV trafficking. Our findings provide mechanistic insights into how FIB-mediated P20 methylation positively regulates systemic trafficking of a subviral agent in plants.

## Teaser

Fibrillarin methylates a subviral protein to facilitate satellite RNA trafficking in plants.

## Introduction

RNA transport is an important and dynamic process for intracellular, intercellular, and systemic communication that plays diverse roles in gene silencing, nutrient allocation, development, and stress responses in plants (Pagliarani and Gambino 2019; Maizel et al. 2020). The cell-to-cell movement of RNAs depends on plasmodesmata (PD) (Sun et al. 2019), whereas systemic transport is enabled by the phloem. Sieve element and companion cells are linked by highly specialized PD, which mediate the selective transport of RNAs via the phloem (Morris 2018; Yang et al. 2019). Endogenous RNA molecules such as small RNAs, tRNAs, ribosomal RNAs (rRNAs), and mRNAs are detectable in phloem exudate and can be transported within the phloem to distant tissues (Kehr and Kragler 2018). Long-distance transport of RNAs in the phloem is mediated by their binding to RNA-binding proteins (RBPs) to form ribonucleoprotein (RNP) complexes (Shi et al. 2020; Tian et al. 2020). Notably, among the mobile mRNAs identified in *Arabidopsis*, nucleolar fibrillarin (FIB) mRNA proved to be graft transmissible (Thieme et al. 2015).

FIB, a member of the S-adenosylmethionine (SAM) methyltransferase (MTase) superfamily, is a highly conserved nucleolar protein. It plays critical roles in epigenetic marks for RNA polymerase I transcription, RNA processing, nuclear architecture, and cellular stress responses (Loza-Muller et al. 2015; Rodriguez-Corona et al. 2015; Zheng et al. 2016). FIB contains 3 functional domains: a glycine- and arginine-rich domain (GAR domain), an RNA-binding domain, and an α-helical domain (Rodriguez-Corona et al. 2015). The MTase domain of FIB resides in its C-terminus, encompassing the conserved RNA-binding and α-helical domains (Rodriguez-Corona et al. 2015). Protein modeling of the 3-dimensional structure of *Arabidopsis thaliana* FIB2 (AtFib2) based on human FIB revealed that the MTase catalytic triad (residues K138, D231, and K260) resides in a pocket formed by the SAM-binding site and RNA-binding domain (Rakitina et al. 2011). FIB is methylated by protein arginine MTases (PRMTs) 1, 6, and 7 (Bedford and Richard 2005), and the arginine methylation of the *N*-terminal GAR domain directs FIB to the nucleus (Shubina et al. 2020). Apart from methylating rRNAs and small RNAs (Rakitina et al. 2011), FIB also triggers Gln-105 methylation of histone H2A in human cells and *Brassica oleracea* (Tessarz et al. 2014; Loza-Muller et al. 2015). Some FIBs have been reported to exert ribonuclease activity in humans, yeasts, and plants (Rodriguez-Corona et al. 2017; Guillen-Chable et al. 2020; Shubina et al. 2020; Smith et al. 2020).

FIB interacts with several plant viral proteins and participates in the diverse infection cycles of plant viruses (Stamm and Lodmell 2019; Xu et al. 2020). Not only does FIB contribute to long-distance transport, as reported for its interactions with groundnut rosette virus (GRV) ORF3 (Kim et al. 2007a, 2007b), rice stripe virus (RSV) P2 (Zheng et al. 2015), and satellite RNA (satRNA) of bamboo mosaic virus (satBaMV) P20, but it also acts in cell-to-cell movement, as described for interactions with barley stripe mosaic virus (BSMV) TGB1 (Li et al. 2018), and contributes to viral accumulation through interactions with the potato virus A (PVA) VPg domain of NIa (Rajamaki and Valkonen 2009) and beet black scorch virus P7a (Wang et al. 2012). Other viral proteins, such as poa semilatent virus (PSLV) TGB1 and grapevine red blotch-associated virus V2, interact or colocalize with FIB in the nucleolus (Semashko et al. 2012; Guo et al. 2015), but the effects of FIB on viral infection in those cases remain unclear.

satRNAs, representing parasites of RNA viruses, are almost exclusively associated with plant viruses and lack appreciable sequence similarity to the genomes of their helper viruses (HVs), yet they depend on HV-encoded replicase and capsid protein (CP) for their replication and encapsidation, respectively (Roossinck et al. 1992; Simon et al. 2004; Hu et al. 2009). satBaMV, a single-stranded positive-sense RNA (Lin and Hsu 1994), encodes a 20 kDa protein (P20) that is dispensable for replication (Lin et al. 1996) but is required for long-distance transport of satBaMV in *Nicotiana benthamiana* (Palani et al. 2006; Vijayapalani et al. 2012). A previous study showed that the *N*-terminal arginine-rich motif (ARM) of P20 is a key determinant of satBaMV RNA binding, self-interaction, and phosphorylation, which regulates cell-to-cell and long-distance movements of satBaMV (Vijayapalani et al. 2012). Moreover, even in the absence of its HV, satBaMV can move autonomously and systemically in *N*. *benthamiana*, with FIB playing a vital role in assisting long-distance satBaMV movement via the formation of a mobile P20–satBaMV–FIB RNP complex (Chang et al. 2016). Silencing of FIB suppresses long-distance satBaMV transport alone, and not that of bamboo mosaic virus (BaMV), in coinfected *N. benthamiana* (Chang et al. 2016). BaMV, the HV of satBaMV, comprises 5 open reading frames (ORFs) that encode replicase, triple gene block protein 1–3 (TGBp1–3), and CP, with TGBp1–3 and CP all being required for cell-to-cell movement (Lin et al. 1994, 2004, 2006; Lan et al. 2010; Lee et al. 2011; Chou et al. 2013).

Here, we explore the molecular function of FIB in systemic trafficking of satBaMV, demonstrating that FIB can directly methylate P20 both in vitro and in vivo. The MTase activity of FIB is biologically significant since MTase-defective mutants display impaired P20 methylation, which reduces satBaMV-binding activity and long-distance transport. Additionally, we also show that FIB-mediated P20 methylation contributes to the trafficking of the P20–FIB complex to PD. Notably, the nucleolar localization of P20 for its methylation is required for autonomous long-distance satBaMV transport in plants. Our study establishes a mechanistic connection between nucleolar FIB-mediated P20 methylation and systemic satBaMV transport in plants.

## Results

### FIB methylates P20 in vitro and binds to satBaMV RNA

**Figure 1.**
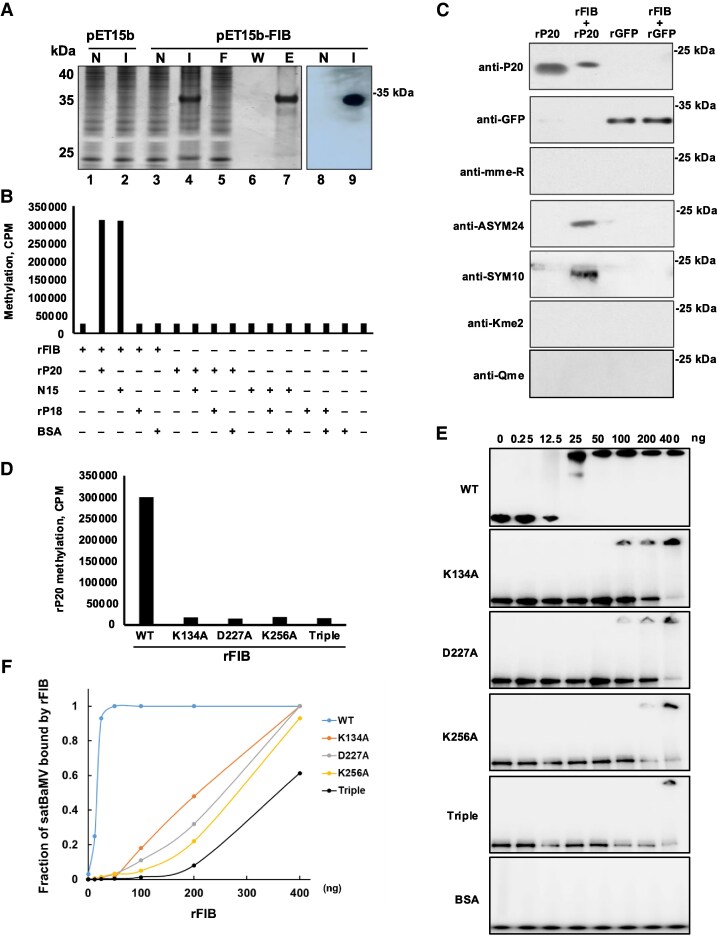
FIB protein methylates P20 and binds to satBaMV RNA. **A)** SDS–PAGE and western blot analysis of expressed and purified rFIB. Total proteins were extracted from *E. coli* harboring pET15b-FIB and analyzed using 12% SDS–PAGE followed by Coomassie blue staining. Lane 4 shows the total protein, while lanes 5 and 6 represent the flow-through and wash fractions (with 20 mm imidazole), respectively. The rFIB was eluted using 200 mm imidazole and appears in lane 7. Marker protein positions are indicated on the left. Western blotting was performed using an anti-FIB antibody (Santa Cruz) to confirm the presence of rFIB in the purified sample (lanes 8 and 9). N: no IPTG; I: IPTG induction; F: flow-through; W: wash; E: elution. **B)** In vitro methylation assay of rP20 by rFIB. To assess FIB-dependent methylation, rP20, rP18, or N15 (a 15-amino acid ARM in the N-terminal region of P20) was incubated with rFIB and [^3^H]-labeled SAM. The level of methylation was determined by measuring radioactivity incorporated into the proteins, expressed as counts per minute (CPM). BSA served as the negative control. The results shown are representative of 3 independent experiments, each performed with 4 replicates. **C)** Western blot analysis of methylated rP20 catalyzed by rFIB. rP20 was incubated with rFIB for in vitro methylation and subsequently analyzed using antibodies specific for various methylation marks: anti-MMA (anti-mme-R, Abcam), anti-SDMA (anti-SYM10, Millipore), anti-ADMA (anti-ASYM24, Millipore), anti-DMK (anti-Kme2, Invitrogen), and anti-MQ (anti-Qme, Millipore). GFP served as a negative control. Three independent experiments were conducted and produced similar results. **D)** In vitro methylation of rP20 by WT and mutant rFIB proteins. To investigate the methylation of rP20 by rFIB WT or MTase mutants, an in vitro methylation assay was performed using both WT rFIB and its MTase mutants. The assay followed the same procedure as described in panel B, comparing the methylation activity between WT and mutant proteins. Data are presented from 1 of 3 independent experiments, each conducted with 4 technical replicates. **E)** EMSA to determine rFIB–satBaMV interaction. Different concentrations of purified rFIB (WT and MTase mutants) were incubated with a ^32^P-labeled satBaMV probe. The RNA–protein complexes were separated on a 4% to 12% polyacrylamide gel and visualized using Phosphor-imager scanning. BSA was used as a control to confirm specific binding. **F)** The binding efficiency of rFIB (WT and mutants) to satBaMV RNA was determined by the fraction of shifted RNA in each lane relative to the amount of bound protein. Representative results from 3 independent experiments with 4 replicates each are shown.

To obtain a substantial quantity of FIB for biochemical analysis, we inserted the His-tagged cDNA of FIB from *N*. *benthamiana* into a T7 expression vector, pET15b, to generate pET15b-FIB. Isopropyl β-D-1-thiogalactopyranoside (IPTG) induction yielded substantial amounts of recombinant FIB (rFIB) (Fig. 1A, lane 4). After Ni^2+^ affinity column purification (Fig. 1A, lane 7) and validation by western blotting using anti-FIB antibody (Fig. 1A, lane 9), we conducted a methylation assay on the purified rFIB to determine if FIB can methylate P20 in vitro. We found that rFIB methylated recombinant P20 (rP20) as well as an N15 peptide (comprising the 15 *N*-terminal amino acids of P20, the ARM), but not a recombinant P18 (rP18) (ARM-deleted P20 mutant) (Tsai et al. 1999) or BSA, indicating that P20 ARM is the specific target for FIB methylation (Fig. 1B). Next, we used various methylation-specific antibodies—including against monomethylarginine (MMA), asymmetric dimethylarginine (ADMA), symmetric dimethylarginine (SDMA) (Blanc and Richard 2017), dimethyllysine (DMK), and methylglutamine (MQ)—to examine rFIB-dependent rP20 methylation. We detected strong signals of methylated rP20 using the ADMA- and SDMA-specific antibodies, but not for MMA, DMK, or MQ antibodies, indicating that P20 primarily undergoes ADMA and SDMA modifications (Fig. 1C).

Previously, the MTase catalytic triad of AtFib2 was identified as K138/D231/K260 (Rakitina et al. 2011). Based on a sequence alignment of FIB from *Arabidopsis* and *N. benthamiana*, we generated single or triple K134/D227/K256 NbFIB2 MTase mutants for an activity assay (Supplementary Fig. S1). As shown in Fig. 1D, only wild-type (WT) rFIB was able to methylate rP20, but the single or triple mutants did not, supporting that the catalytic triad of FIB MTase residues is essential for P20 methylation.

Since we have previously shown that FIB forms RNP complexes with P20 and satBaMV RNA (Chang et al. 2016), we wondered if FIB could directly bind to satBaMV RNA. Using serial amounts of rFIB in an electrophoretic mobility shift assay (EMSA), we observed satBaMV RNA mostly in bound form upon increasing rFIB levels to a protein/RNA weight ratio of 16:1, which resulted in a rapid transition of the satBaMV RNA to a complete FIB–satBaMV RNP (Kd ∼50 nm) (Fig. 1E). In contrast, the rFIB K134A mutant bound satBaMV RNA less efficiently than WT rFIB, with a Kd of ∼140 nm, and the rFIB D227A and K256A mutants (Kd ∼350 nm), respectively, proved even less potent (Fig. 1E). The rFIB triple mutant exhibited the lowest binding affinity for satBaMV, with a Kd value of ∼1,230 nm. In contrast, BSA did not bind to satBaMV RNA under the same conditions (Fig. 1E). The Hill coefﬁcients for satBaMV-binding by rFIB or its mutants range from 2.3 to 4.2, implying that binding of satBaMV RNA by rFIB occurs in a positively cooperative manner (Fig. 1, E and F). Together, these data support that rFIB binds directly to satBaMV RNA and that its MTase domain is important for this interaction.

### FIB's MTase catalytic triad is required for long-distance satBaMV trafficking

**Figure 2.**
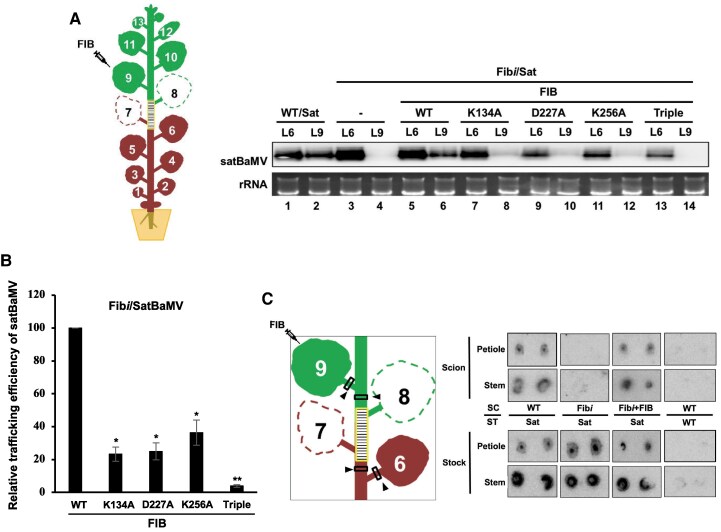
Complementation of autonomous long-distance satBaMV trafficking by WT and mutant FIBs. **A)** Accumulation of satBaMV RNA in WT or Fib*i* scions overexpressing WT or mutant FIB proteins. WT or Fib*i* scions overexpressing either WT NbFIB2 or its MTase mutants were grafted onto *N. benthamiana* transgenic stocks expressing satBaMV. L9 of the scions was infiltrated with *Agrobacterium tumefaciens* strain C58C1 carrying either WT NbFIB2 or mutant variants. At 9 DPA, L6 and L9 were harvested for RNA gel blot analysis to assess satBaMV accumulation. **B)** Relative trafficking efficiency of satBaMVs across the grafting union. To quantify the trafficking efficiency of satBaMVs, RNA gel blots from 3 independent experiments were analyzed using ImageJ software and normalized to rRNA levels. Trafficking efficiency was defined as the ratio of satBaMV signals in L6 to L9, with the efficiency of WT FIB set to 100%. Data are presented as mean ± SD, and statistical significance was determined by Student's *t*-test (* *P* < 0.05, ** *P* < 0.005). **C)** Tissue blotting to detect satBaMV accumulation. Stem and petiole tissues from the scions and transgenic stock (indicated by arrowheads) were imprinted onto membranes and probed with a satBaMV-specific probe (Lin et al. 1996) to detect satBaMV accumulation. Three independent biological samples from A and C, each involving 3 plants, yielded consistent results.

We previously demonstrated that FIB is crucial for autonomous systemic transport of satBaMV in *N. benthamiana* (Chang et al. 2016). Therefore, we investigated if FIB MTase activity is important to rescue satBaMV systemic transport in Fib*i* scions—which have approximately 40% residual FIB levels—when grafted onto the satBaMV-transgenic *N. benthamiana* (Chang et al. 2016). As previously described, grafting assays demonstrated the systemic movement of satBaMV from satBaMV-transgenic stock exclusively to leaf 9 (L9) of WT scions 12 days after grafting (Chang et al. 2016). Thus, we routinely sample L6 and L9 for assays under identical experimental conditions. Consistent with the previous report (Chang et al. 2016), we observed trafficking of satBaMV from the satBaMV-transgenic stocks into WT scions, but not into Fib*i* scions (Fig. 2A, lanes 2 and 4, respectively). In contrast, BaMV-eGFP accumulated significantly in L9 of both WT and Fib*i* scions (Supplementary Fig. S2C), revealing that systemic BaMV movement is FIB independent. Interestingly, northern blot analysis revealed a substantial amount of satBaMV in L9 of Fib*i* scions when coexpressed with WT FIB at 9 days post agroinfiltration (DPA) (Fig. 2A, lane 6). However, the accumulations of satBaMV in L9 of Fib*i* scions hosting NbFIB2 MTase single mutants, K134A, D227A, or K256A, were only 20% to 40% those of WT (Fig. 2, A and B), with an even more pronounced reduction to less than 10% in the NbFIB2 triple mutant (Fig. 2A, lane 14; Fig. 2B).

To determine whether satBaMV is trafficked via vascular tissues, we assayed the stems and petioles adjacent to the scion graft site by tissue blotting, which uncovered satBaMV accumulations in Fib*i* scion stems and petioles upon FIB complementation at 9 DPA (Fig. 2C). In contrast, no satBaMV was detected in the Fib*i* scion stem or petiole samples in the absence of FIB expression (Fig. 2C). Thus, transient FIB expression supports the transportation of satBaMV from transgenic satBaMV stock to Fib*i* scions, with the FIB MTase domain being crucial for satBaMV transport.

### Residue R5 of P20 ARM is methylated by FIB in *N*. *benthamiana*

**Figure 3.**
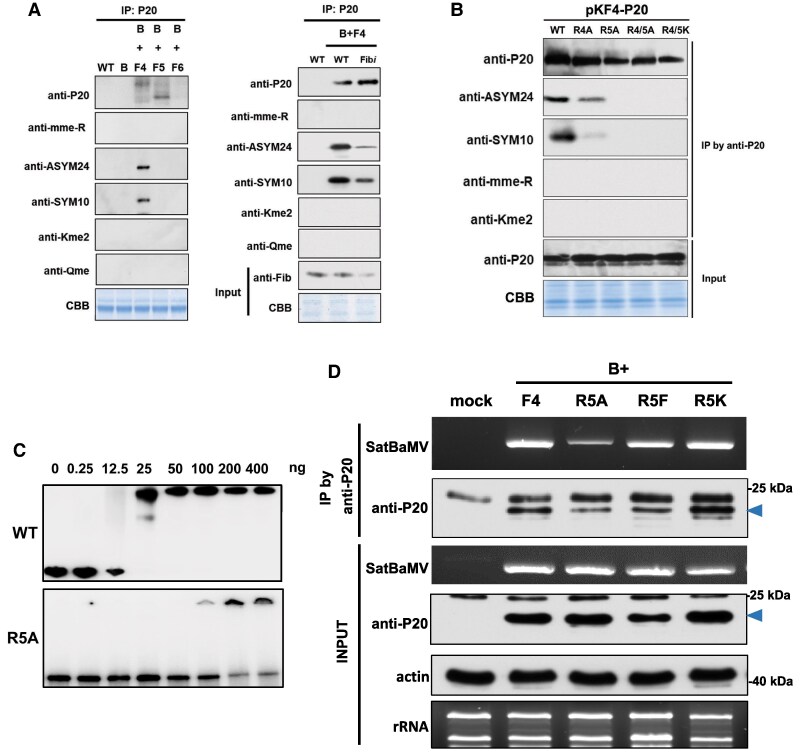
FIB-mediated arginine methylation of P20. **A)** Detection of methylated P20 in vivo by western blot analysis. The pKF4 (F4), pKF5 (F5), or pKF6 (F6) constructs were coinfiltrated with pKB (B) in WT or Fib*i* plants. Total proteins were immunoprecipitated by anti-P20 antibody, followed by detection of P20 methylation and its variants using anti-mme-R, anti-SYM10, anti-ASYM24, anti-Kme2, and anti-Qme antibodies, as described in Fig. 1. Coomassie Brilliant Blue (CBB) staining for Rubisco was used as input protein control prior to IP. **B)** Detection of methylated P20 WT or mutants in vivo. The pKF4 construct or respective mutants was infiltrated into WT *N. benthamiana* leaves, immunoprecipitated, and analyzed by western blot to detect P20 methylation, as described in A. The data presented are representative of 3 independent experiments. **C)** EMSA of the rP20− or R5A mutant interactions with satBaMV. Purified WT or mutant rP20-R5A at the indicated amounts was incubated with satBaMV riboprobe to analyze protein–RNA interaction, as described in Fig. 1E. Representative results from 3 independent experiments are shown. The WT blot image has been reused from Fig. 1E, as it was obtained under identical experimental conditions. **D)** Binding affinities of P20 or its mutants to satBaMV in the BaMV and satBaMV coinfected *N*. *benthamiana*. The coinfected plant samples were immunoprecipitated with anti-P20 antibody, and the immunoprecipitated RNA was extracted and subjected to RT-qPCR to detect the presence of satBaMV. The arrowheads indicate the expected P20 protein.

To analyze P20 methylation in vivo, we coinfiltrated BaMV (pKB, an infectious clone of BaMV) (Liou et al. 2014) into *N. benthamiana* leaves with WT satBaMV (pKF4, an infectious clone of satBaMV) (Liou et al. 2014), a P20 ARM deletion mutant (pKF5, encoding P18 protein), or a P20 frameshift mutant (pKF6) (Lin et al. 1996). Immunoprecipitation (IP) of anti-P20 serum at 5 DPA revealed P20 and P18 protein accumulation in plants coinfiltrated with pKF4 or pKF5, respectively (Fig. 3A, left panel), but only the ADMA and SDMA signals of P20 were detectable by specific antibodies (Fig. 3A, left panel). However, the ADMA and SDMA signals of P20 were significantly reduced in Fib*i* lines compared to WT plants (Fig. 3A, right panel), indicating that the ADMA- and SDMA-type methylations of P20 are FIB dependent.

To identify the FIB-dependent methylation sites of P20, we performed an in vitro methylation assay by incubating rFIB in the presence of the methyl group donor SAM and N15 peptide (corresponding to the P20 ARM). By conducting tandem mass spectrometry (MS/MS) analysis, we found that the N15 ARM peptide was monomethylated in vitro by rFIB at both the R4 and R5 residues (Supplementary Fig. S3). To confirm P20 methylation in vivo, we generated single or double P20 R4/R5 mutants and agroinfiltrated them into *N*. *benthamiana*. After IP with the anti-P20 serum, western blotting revealed a stable accumulation of P20 and its mutant variants expressed by satBaMV. However, the ADMA- and SDMA-type methylations of P20 were detected solely in the WT and pKF4-R4A mutant, but not in the pKF4-R5A, R4/5A, or R4/5K mutants (Fig. 3B), evidencing that the R5 residue of the P20 ARM is methylated in vivo.

Given that the R5 residue of the P20 ARM is methylated by FIB both in vitro and in vivo, we wondered if that residue is important for the binding of P20 to satBaMV. EMSA with purified rP20 or rP20-R5A showed that at least 100 ng of rP20-R5A was needed to retard the mobility of the satBaMV probe compared to 25 ng of rP20 (Fig. 3C). The Kd value for binding of rP20-R5A to satBaMV is 150 nm compared to 32 nm for rP20, indicating that the R5A mutation of P20 substantially impairs satBaMV binding in vitro. To further determine the effects of P20 methylation on satBaMV binding in vivo, satBaMV RNA from WT P20- or P20-R5-mutated RNP complexes was purified by RNP IP (RIP) in *N*. *benthamiana* (Fig. 3D). After IP with the anti-P20 serum, the immunoprecipitated RNA was extracted and subjected to RT-PCR analysis. SatBaMV RNAs were detected in all precipitates of satBaMVs harboring WT P20, nonmethylated P20 (R5A and R5K), and methylation-mimic P20 (R5F) (Fig. 3D), indicating that the methylation status of R5 does not affect its RNA-binding activity in vivo.

### The R5 residue of P20 is critical for systemic satBaMV transport

**Figure 4.**
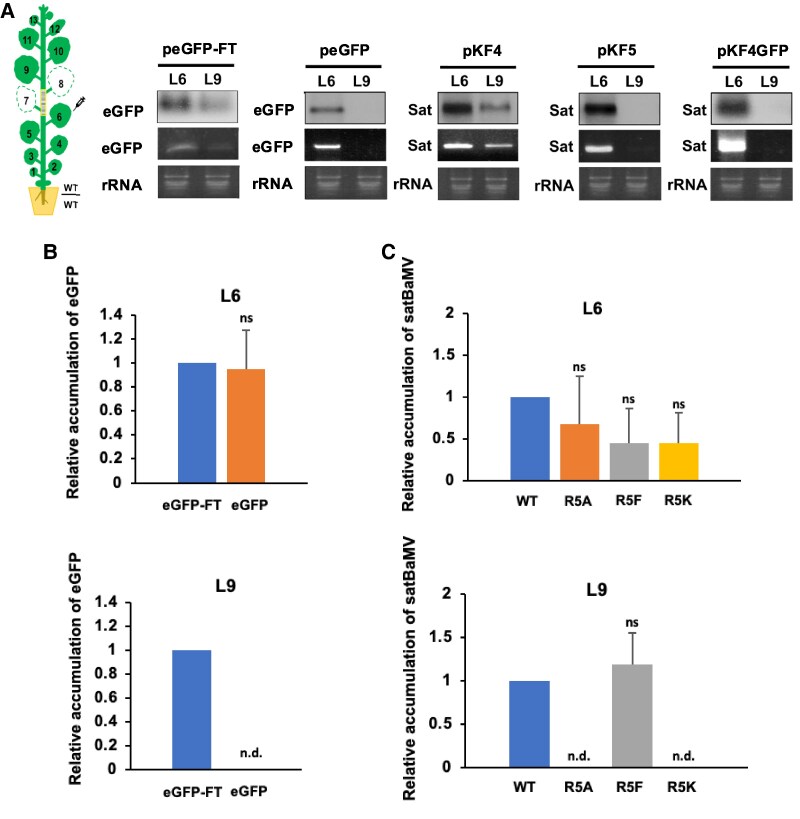
The 5th arginine in the P20 ARM is critical for long-distance satBaMV trafficking. **A)** Northern blot analysis of long-distance trafficking assay. The grafting experiment was conducted on 40-day-old WT-to-WT (WT/WT) engrafted *N. benthamiana* in which the tested mRNA was agroinfiltrated into stock leaves (L6), and its mobility into the engrafted scion (L9) was measured at 9 DPA. The accumulation of satBaMV (Sat) (from pKF4, pKF5, and pKF4GFP), eGFP-FT, and eGFP RNAs was analyzed by RNA gel blotting and RT-PCR. rRNA served as a loading control. Specific RNAs were detected using hybridization probes complementary to part of the target sequence. **B, C)** Statistical analysis of eGFP-FT, eGFP, satBaMV, and R5 mutant accumulation in grafted L6 stock and L9 scion leaves by RT-qPCR. Values are normalized to the WT sample, with actin as an internal control. Three independent biological replicates, each comprising at least 3 plants, produced similar results. Data are represented by the mean ± SD from 3 experiments and were analyzed using Student's *t*-test (n.d., not detectable; ns, not significant).

We routinely use satBaMV-transgenic stock to monitor the autonomous trafficking of satBaMV into grafted scions or vice versa. To establish a simple system for mRNA mobility assays, we grafted WT scions onto WT *N. benthamiana*, agroinfiltrated mRNA into stock leaves (L6), and measured mRNA trafficking into the scion (L9) at 9 DPA. As expected, northern blotting and RT-qPCR revealed mobile GFP-tagged FLOWERING LOCUS T (FT) RNA, but not GFP RNA, trafficked into scion L9 leaves (Fig. 4, A and B). In parallel, we also confirmed the movement of satBaMV from inoculated L6 leaves into L9 of scions. In contrast, neither the pKF5 mutant nor pKF4GFP (where P20 is replaced with GFP) moved into L9 (Fig. 4A). Thus, our simple WT-onto-WT grafting assay enabled us to study autonomous mRNA transport into *N. benthamiana*.

Accordingly, we agroinfiltrated WT satBaMV or P20-R5 mutants into the L6 leaves of WT *N. benthamiana* engrafted with WT scions (Fig. 4A). From 3 independent experiments, both satBaMV with WT P20 and the methylation-mimic P20-R5F mutant were transported to L9 leaves, while systemic transport of satBaMV was completely abolished with nonmethylatable alanine (P20 R5A) and lysine mutant (P20 R5K) by RT-qPCR analysis at 9 DPA (Fig. 4C). This was observed despite the accumulation of P20 mutants in L6 leaves showing no significant difference in levels compared to WT satBaMV (Fig. 4C). These grafting experiments demonstrate that the methylation of R5 residue of P20 is essential for satBaMV to move systemically across the graft site.

### FIB mediates the plasmodesmal targeting of P20

**Figure 5.**
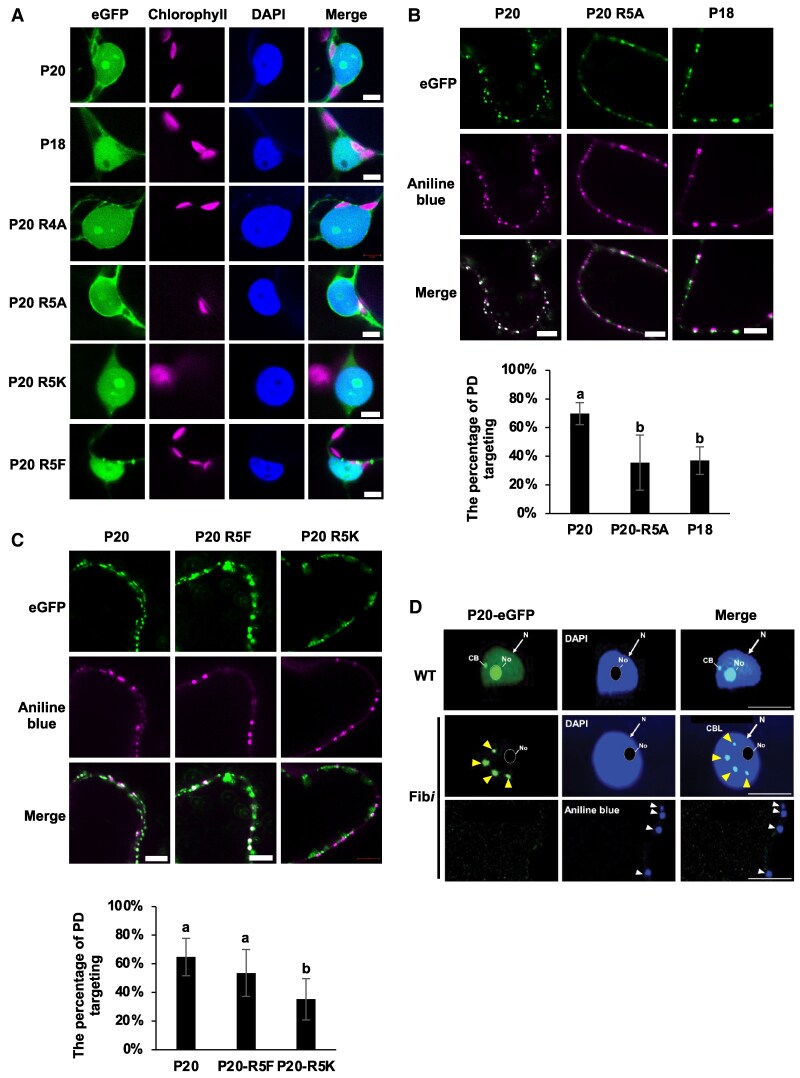
Subcellular localization of P20 and its mutants in *N*. *benthamiana* cells. **A)** Confocal images showing the localization of P20 and its mutant proteins in *N. benthamiana.* Leaves were infiltrated with *Agrobacterium* carrying pBIN61-P20-eGFP, P18-eGFP, P20 R5A-eGFP, P20 R5K-eGFP, or P20-R5F-eGFP. At 2 DPA, leaves were harvested and stained with DAPI to visualize nuclei. Imaging was performed using a Leica STELLARIS 8 confocal microscope. Scale bars: 5 µm. **B, C)** Localizations of P20-eGFP and its mutants at PD. The localization of P20-EGFP and its mutants at PD was visualized using aniline blue staining. The percentage of PD targeting was calculated as the number of colocalized P20 or mutant signals with aniline blue, divided by the total number of aniline blue signals along the cell wall. Colocalization was analyzed from 9 **(B)** and 19 **(C)** images, respectively, using ANOVA with Tukey's test. Different letters indicate significant differences (*P* < 0.05). The error bars represent the ±SD of 3 independent experiments. **D)** Subcellular localization of P20-eGFP in WT and Fib*i* plants. Localization of P20-eGFP was examined in epidermal cells of infiltrated leaves from WT or Fib*i N*. *benthamiana* leaves stained with DAPI or aniline blue at 2 DPA. N: Nucleus; No: Nucleolus; CB: Cajal body; CBL: Cajal body-like structures. Arrowheads in the middle or lower panels of D indicate the CBL structures (yellow) or PD (white), respectively. Scale bars: 5 µm.

Methylation of the arginine residue of FIB protein controls its nuclear import and nucleolar accumulation (Guillen-Chable et al. 2020; Shubina et al. 2020; Smith et al. 2020). To examine if P20 methylation alters its subcellular localization, we expressed eGFP-tagged P20 and mutant variants in *N. benthamiana* and examined protein localization by confocal microscopy. Leaves were stained with 4′,6-diamidino-2-phenylindole (DAPI) or aniline blue to visualize nuclei or PD, respectively. Confocal microscopy revealed that P20-eGFP and the R4A and R5K mutants accumulated in the nucleus, with particular enrichment in the nucleolus, and in the cytoplasm (Fig. 5A). In contrast, the R5A substitution mutant reduces the nucleoli targeting, and the ARM-deleted P18 and R5F substitution mutants failed to localize in the nucleoli (Fig. 5A), indicating that the nucleolar localization of P20 may be affected by R5 modification.

In addition to accumulating in the nucleolus, nucleus, and cytoplasm, P20-eGFP also formed punctate structures at PD (Fig. 5, B and C). Analysis of the signal distribution of P20-eGFP and the PD marker aniline blue along the cell wall (Supplementary Fig. S4) revealed that the PD targeting efficiencies of WT P20-eGFP and the R5F methylation-mimic mutant were about 65% and 54%, respectively (Fig. 5C). In contrast, the nonmethylated P20-eGFP mutants P18 (37%), R5A (36%), and R5K (35%) exhibited significantly reduced colocalization with aniline blue signal (Fig. 5, B and C), indicating that methylation of the R5 residue in P20 enhances the efficiency of its PD targeting.

Furthermore, we used Fib*i* plants to investigate if the transport of P20-eGFP into the nucleolus and PD targeting are FIB dependent. In Fib*i* plants, P20-eGFP predominantly localized to multiple Cajal body-like structures within the nuclei (Fig. 5D), resembling the phenotype observed for GRV ORF3 in Fib*i* plants (Kim et al. 2007a). Additionally, the PD targeting of P20-eGFP was significantly reduced in Fib*i* plants (Fig. 5D). Together, these results indicate that both nucleolar and PD targeting of P20 are dependent on FIB.

### P20 facilitates FIB targeting to PD

Given that FIB is a conserved nucleolar protein, we wondered how it is trafficked intracellularly to PD during satBaMV infection. To address this question, we coexpressed mCherry-NbFIB2 with P20-eGFP or P20 R5A-eGFP mutant in *N*. *benthamiana* by agroinfiltration followed by confocal microscopy. At 2 DPA, approximately 70% P20-eGFP puncta were colocalized with mCherry-NbFIB2 at PD along the cell periphery (Supplementary Fig. S5, A, B, and D). In contrast, the colocalizations of P20 R5A-eGFP and mCherry-NbFIB2 at PD were significantly reduced to about 25% (Supplementary Fig. S5, A, C, and D), indicating that PD targeting of the P20–FIB complex is regulated by P20 methylation.

Next, we examined if satBaMV infection triggers the nuclear-cytoplasmic trafficking of FIB. To do so, we inoculated the leaves of transgenic *N*. *benthamiana* expressing BaMV RNA-dependent RNA polymerase (ORF1-HA, Lee et al. 2011) with transcripts of either WT satBaMV or the F5 mutant (Supplementary Fig. S6). Five days post inoculation, we expressed eGFP-NbFIB2 in the same leaves by agroinfiltration and examined its subcellular localization by confocal microscopy at 2 DPA (Supplementary Fig. S6A). In plants inoculated with WT F4 satBaMV, we detected punctate signals of eGFP-NbFIB2 at the cell periphery and colocalization with aniline blue signals, despite substantial amounts of eGFP-NbFIB2 also accumulating in the nucleolus and nucleoplasm (Supplementary Fig. S6B). In contrast, plants inoculated with the ARM-deleted mutant, satBaMV F5, or mock treatment showed minimal colocalization of eGFP-NbFIB2 with aniline blue signal (Supplementary Fig. S6B). Co-IP and western blotting further demonstrated that P18, unlike P20, failed to interact with FIB (Supplementary Fig. S6C). These results support that the P20 ARM mediates the relocalization of FIB from the nucleolus to PD during satBaMV infection.

**Figure 6.**
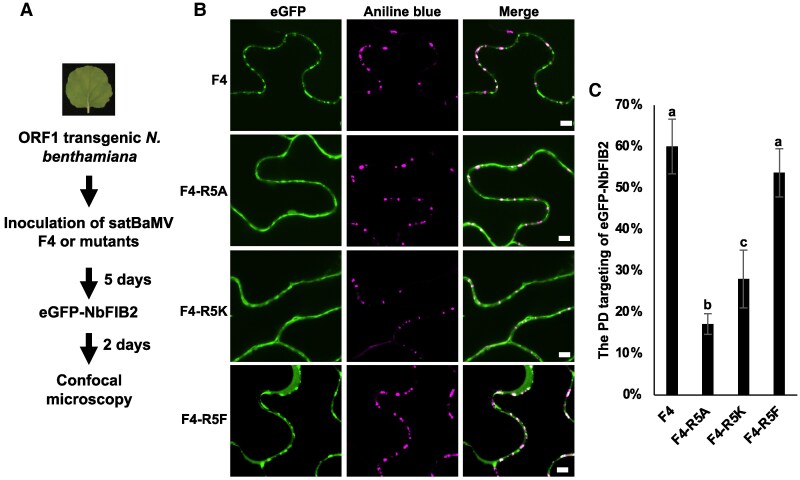
satBaMV-triggered relocalization of FIB. **A)** Flowchart of the subcellular localization procedure for eGFP-NbFIB2. A flowchart outlining the procedure used to visualize the subcellular localization of eGFP-NbFIB2 following inoculation with satBaMV F4 WT or mutants. **B)** Subcellular localization of eGFP-NbFIB2 after satBaMV F4 or mutant inoculation. Leaf sections were stained with aniline blue and examined using a Zeiss LSM880 Airyscan confocal microscope. Scale bars represent 5 µm. **C)** PD targeting of eGFP-NbFIB2 triggered by satBaMV or its mutants. The distribution of eGFP-NbFIB2 along the cell wall, particularly at PD, was analyzed using ZEN 3.7 blue software. The colocalization of eGFP-NbFIB2 at PD was quantified and statistically analyzed by ANOVA followed by Tukey's test. Significant differences between groups are indicated by different letters (*P* < 0.05, *n* = 7). The error bars represent the ±SD of 3 independent experiments.

To further investigate the effect of P20-R5 methylation on the PD targeting of FIB, we examined the colocalizations of eGFP-NbFIB2 with the infections of satBaMV F4 P20-R5 mutants (Fig. 6A), including F4-R5A, F4-R5K, and F4-R5F. Our results showed that both WT F4 and methylation-mimic F4-R5F mutant induced about 60% of eGFP-NbFIB2 to localize at PD (Fig. 6, B and C). In contrast, the 2 nonmethylated P20 mutants, R5A and R5K, triggered only 18% and 20% eGFP-NbFIB2 localization at PD, respectively (Fig. 6, B and C), indicating that the P20-R5 methylation plays a critical role in facilitating the accumulation of eGFP-NbFIB2 at PD.

### Methylated P20 in the nucleus is required for systemic satBaMV transport

**Figure 7.**
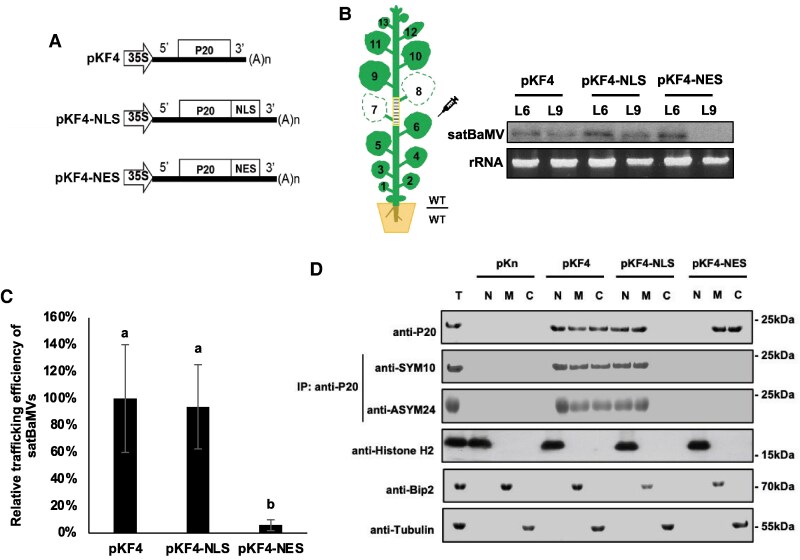
A nuclear localization of P20 is crucial for long-distance satBaMV trafficking. **A)** Schematic diagram showing pKF4 and its derivative constructs. The physical maps of pKF4, pKF4-NLS, and pKF4-NES in binary vector are shown. **B)** Northern blot analysis of pKF4 and its derivatives in WT/WT-grafted *N. benthamiana* plants. Leaf 6 (L6) of WT/WT engrafted *N. benthamiana* plants was infiltrated with *Agrobacterium* carrying pKF4, pKF4-NLS, or pKF4-NES. At 9 DPA, L6 and L9 were harvested for northern blotting. **C)** Trafficking efficiencies of satBaMV and its mutants. The trafficking efficiency of satBaMV RNAs from panel B was quantified and represented as mean ± SD from 4 independent experiments, with pKF4 set as 100%. Statistical analysis was conducted using ANOVA followed by Tukey's test. Different letters indicate statistically significant differences (*P* < 0.05, *N* = 4). **D)** Cell fractionation analysis of methylated P20. Total proteins (T) were extracted from nuclei-depleted (C), nuclei-enriched (N), and membrane-enriched (M) fractions of leaves infiltrated with pKF4 and its derivatives at 7 DPA. Dimethylated P20 was detected using anti-SYM10 and anti-ASYM24 antibodies. Tubulin, Bip2, and Histone H2 were used as cytosolic, membrane, and nuclear markers, respectively. Representative data from 4 independent experiments are shown.

To further explore if the nuclear localization of P20 is essential for long-distance satBaMV transport, we introduced a nuclear localization signal (NLS) or nuclear export signal (NES) (Sun et al. 2013) into the C-terminus of P20 to generate the satBaMV mutants with nucleus-enriched P20 (pKF4-NLS) and nucleus-empty P20 (pKF4-NES), respectively (Fig. 7A). Confocal microscopy revealed that P20-NLS-eGFP localized exclusively in the nucleus, including the nucleolus, while P20-NES-eGFP accumulated in the cytoplasm, excluding the nucleus (Supplementary Fig. S7A). Additionally, N15-eGFP accumulated in the nuclei, whereas P18-GFP was excluded from the nucleolus (Supplementary Fig. S7B). To assess their long-distance transport in WT-onto-WT engrafted *N*. *benthamiana* plants (Fig. 7B), pKF4-NLS satBaMV, similar to satBaMV F4, substantially accumulated in both inoculated leaves and L9 systemic leaves (Fig. 7, B and C). In contrast, pKF4-NES satBaMV failed to accumulate in L9 leaves (Fig. 7, B and C). These results demonstrate that the nuclear localization of P20 is crucial for long-distance transport of satBaMV in plants.

To determine where P20 methylation occurs, we separated the nuclear, cytoplasmic, and P100 membrane-enriched fractions from *N. benthamiana* leaves infiltrated individually with pKF4, pKF4-NLS, pKF4-NES, or empty vector pKn. Subsequent western blotting revealed methylated P20 in the nuclear, cytoplasmic, and P100 membrane-enriched fractions. Methylated P20-NLS was exclusively observed in the nuclear and P100 membrane-enriched fractions (Fig. 7D). In contrast, P20-NES was predominantly enriched in the cytoplasmic and P100 membrane-enriched fractions, but in neither case was the respective P20-NES protein methylated (Fig. 7D). Thus, these results indicate that P20 is methylated in the nucleus.

## Discussion

**Figure 8.**
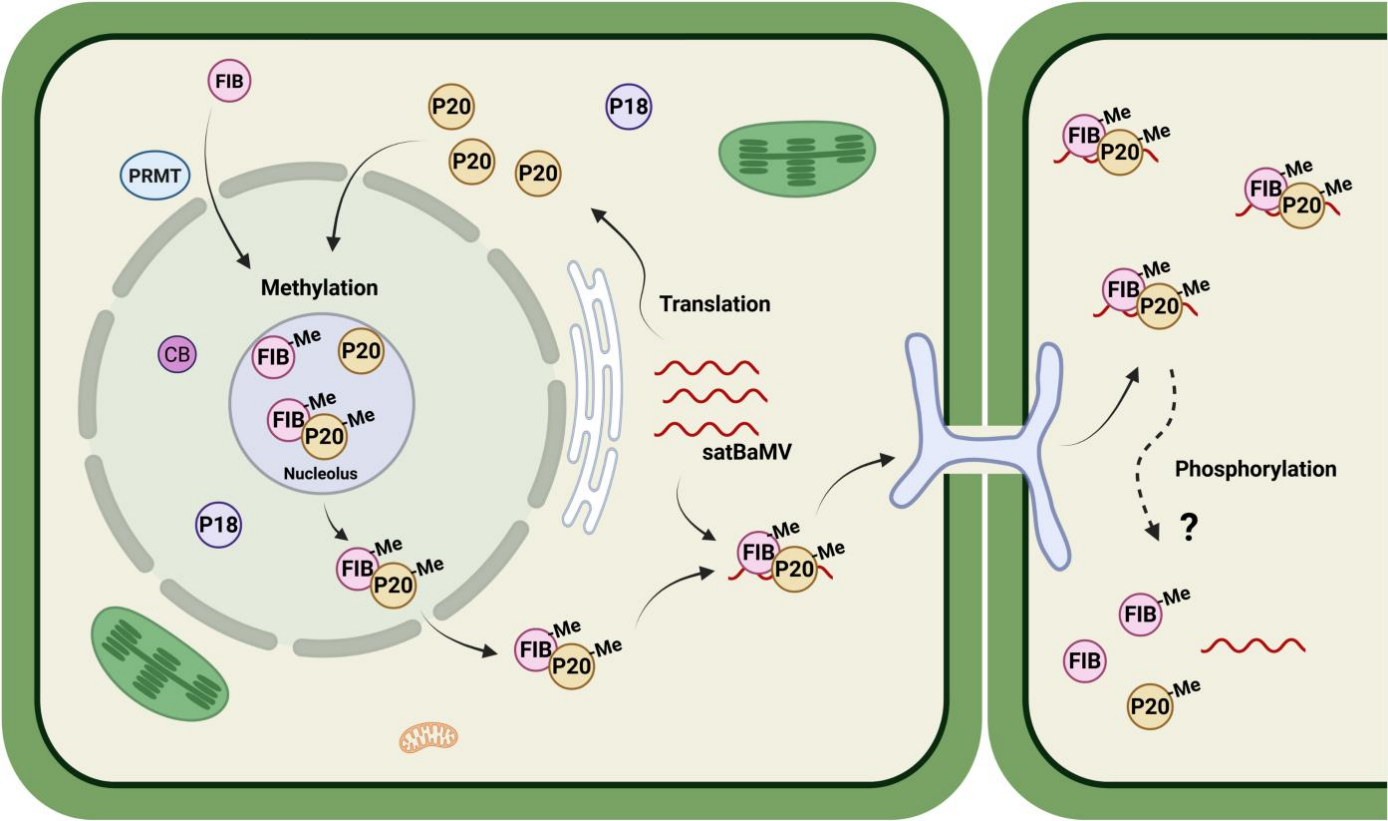
Model of FIB-mediated P20 methylation in facilitating satBaMV trafficking. Upon entry into host cells, satBaMV-encoded P20 is translated by host ribosomes in the cytoplasm. P20 contains a nucleolar localization signal in its ARM that directs it to the nucleolus and Cajal bodies, where it interacts with the FIB protein. FIB is methylated by PRMTs in the cytoplasm (Blanc and Richard 2017) before being trafficked through the nucleus pore complex to Cajal bodies and the nucleolus, where it mediates P20 methylation. Once methylated, the FIB•P20 protein complex translocates to the cytoplasm, where it associates with satBaMV RNA to form a FIB–satBaMV–P20 RNP complex. This RNP complex is directed to PD for cell-to-cell movement. In the neighboring cell, P20 phosphorylation may regulate the fate of the RNP complex. In contrast, P18 accumulates in the nucleus—excluding the nucleolus—and cytoplasm but fails to form an RNP complex with satBaMV, thereby hindering its transport. Me, arginine methylation; CB, Cajal body.

Accumulating evidence indicates that RNA viruses can transport their viral proteins into the nuclei of host cells to recruit nuclear proteins for their movement in infected plants (Decle-Carrasco et al. 2021). Among such viral proteins, BSMV TGBp1 and GRV ORF3 can sequester host-derived FIB into their viral RNP complexes for cell-to-cell movement and/or systemic infection (Kim et al. 2007b; Li et al. 2018; Decle-Carrasco et al. 2021). Moreover, FIB also participates in the formation of satBaMV RNP complexes for autonomous trafficking (Chang et al. 2016). However, how FIB regulates satBaMV movement in plants remains unclear. Based on our findings, we propose a model in which FIB-dependent P20 methylation is responsible for autonomous and systemic satBaMV movement in planta (Fig. 8). Upon satBaMV infection, newly translated P20 is trafficked directly into the nucleolus where it interacts with FIB that methylates it at the R5 residue (Fig. 3). Methylated P20 then triggers the trafficking of FIB together with P20 from the nucleolus into the cytosol, binding directly to satBaMV RNA to form P20–satBaMV–FIB RNP complexes. Consequently, the P20 directs the moving RNP complex to PD (Fig. 5, B and C; Fig. 6, B and C; and Supplementary Figs. S4 and S5), enabling cell-to-cell movement and subsequent systemic movement.

In this study, we demonstrate that rFIB can bind satBaMV RNA directly (Kd ∼50 nm, Fig. 1, E and F). Notably, its binding affinity is quite similar to that of rP20 with satBaMV (Kd = 32 nm, Fig. 3C) (Tsai et al. 1999; Vijayapalani et al. 2012). Thus, satBaMV tightly associates with both FIB and P20 to generate an RNP complex responsible for movement. Interestingly, the RNA-binding ability of rNbFIB was dramatically reduced when we substituted the residues of the NbFIB2 MTase catalytic triad with alanine (Fig. 1F). Given that the NbFIB2 catalytic triad residues are located in sequence stretches corresponding to 2 RNA-binding sites of AtFIB2 (Supplementary Fig. S1) (Rakitina et al. 2011), our findings reveal dual biochemical roles for the FIB MTase catalytic triad in both RNA binding and methylation activity (Fig. 1, D to F). Moreover, we show that the ability of the FIB catalytic triad with P20 methylation is also required for autonomous long-distance satBaMV trafficking based on our grafting and complementation analyses (Fig. 2). FIB MTase methylates P20 at the R5 residue in vivo to generate ADMA and SDMA signals (Fig. 3, A and B), although the N15 peptide was monomethylated in vitro by rFIB at both the R4 and R5 residues (Supplementary Fig. S3). This discrepancy in arginine methylation of P20 protein and N15 peptide may be due to the various catalytic activities of FIB in vitro and in vivo or to differential protein substrates. Thus, we suggest that R5 of the P20 ARM is the key residue for FIB-dependent P20 methylation, which is responsible for the formation of the satBaMV–P20–FIB RNP complex and its systemic movement in planta.

Previous immunoelectron microscopy has revealed that P20 accumulates in the nucleoli, nuclei, and cytoplasm of cells coinfected with BaMV and satBaMV (Palani et al. 2009), but no NLS signal was found for P20 using 2 online NLS predictors, i.e. NLS mapper and NLStradamus (Kosugi et al. 2009; Nguyen Ba et al. 2009). Moreover, we conducted a yeast 2-hybrid analysis and found that P20 does not interact with the nuclear transporters importin α1 and α2 (Supplementary Fig. S8), indicating that nuclear import of P20 may not operate via an importin α-dependent pathway (Goldfarb et al. 2004). In fact, our confocal microscopic analysis did not uncover a nucleolar localization for P18-eGFP (Fig. 5A and Supplementary Fig. S7B), whereas N15-eGFP does accumulate there (Supplementary Fig. S7B), supporting that the first 15 amino acids of P20 represent the nucleolar localization sequence for nucleolar targeting. By studying pKF4-NLS and pKF4-NES infection in *N. benthamiana*, we also detected ADMA or SDMA signals for P20 and P20-NLS in the nuclear and membrane fractions (Fig. 7D), whereas the cytoplasmic- and membrane-enriched P20-NES was nonmethylated (Fig. 7D). Accordingly, we found no nucleolar localization for P20-eGFP and diminished P20 methylation in Fib*i N*. *benthamiana* (Figs. 3A and 5D). Taken together, these results indicate that P20 is methylated by FIB MTase in the nucleus and presumably more specifically in the nucleolus.

In eukaryotes, arginine methylation is catalyzed by PRMTs (Lorton and Shechter 2019), which may alter the charge distribution, shape, and hydrophobicity of the amino acid side chain, thereby modulating protein–protein and protein–nucleic acid interactions responsible for diverse cellular processes (Lorton and Shechter 2019). We found that PD targeting of P20-eGFP was diminished in the R5A, R5K, and P18 nonmethylated mutants but remained unaffected in the R5F methylation-mimic mutant (Fig. 5, B and C), supporting that R5 methylation in the P20 ARM regulates its intracellular localization. Several previous reports have also shown that arginine methylation in the ARM of viral proteins regulates their subcellular localizations and functions. For example, the ARM of human immunodeficiency virus type 1 (HIV-1) Tat protein is methylated by PRMT6 to inhibit nucleolar retention of the viral protein (Fulcher et al. 2016). Similarly, PRMT6 methylates the ARM of HIV Rev protein to diminish its viral RNA-binding ability and to inhibit Rev-mediated viral RNA export from the nucleus to the cytoplasm (Invernizzi et al. 2006). In addition to our previous findings that the ARM of P20 exerts multiple functions, including in RNA binding, self-interaction, and phosphorylation to assist satBaMV movement (Palani et al. 2006; Vijayapalani et al. 2012), herein, we report an additional role for it in facilitating FIB for nucleocytoplasmic shuttling, assembling movement of the RNP complex, and targeting to PD. The autonomous systemic movement of satBaMV was abolished upon deleting the P20 ARM (pKF5, Fig. 4A) and in P20 R5A and R5K mutants (Fig. 4C) due to their inability to be methylated by FIB MTase (Fig. 3B). However, this did not impair their in vivo RNA binding with satBaMV (Fig. 3D), indicating that P20-R5 methylation, rather than its RNA binding activity, plays a critical role in satBaMV systemic movement. Moreover, coexpression of FIB and P20 (Supplementary Fig. S5) or with WT satBaMV infection (Fig. 6 and Supplementary Fig. S6) triggered FIB targeting to PD and promoted colocalization of P20 and FIB at PD. This facilitation of FIB trafficking to PD was reduced during infections with satBaMV F5 or the F4-R5A and F4-R5K mutants but remained unaffected in infection with methylation-mimic F4-R5F mutants (Fig. 6). These findings suggest that only methylated P20 can trigger the colocalization of FIB with P20 at PD. Although our study was conducted primarily in mesophyll cells, we recognize that viral RNA trafficking dynamics and FIB functions may differ significantly in phloem cells, which are specialized for long-distance viral RNA movement. Nonetheless, several studies have highlighted the essential role of FIB in the systemic movement of plant viruses by modulating its subcellular localization. For example, FIB relocalization has been previously observed in association with the c-RNP complex of GRV with ORF3, at PD with the TGB1–RNP complex of BSMV (Kim et al. 2007b; Li et al. 2018) and with 2b protein of cucumber mosaic virus (Zhang et al. 2025). These findings collectively support the notion that FIB translocation to PD is required for efficient long-distance movement of diverse viruses, even though all localization studies thus far have been performed in mesophyll cells.

In addition, only WT and pKF4-NLS satBaMV with detectable ADMA or SDMA signals could move systemically (Fig. 7, B and D). In contrast, pKF4-NES satBaMV with nonmethylated P20 could not undergo long-distance trafficking (Fig. 7, B to D). Taken together, these results indicate that FIB-dependent methylation of P20 in the nucleolus triggers their transport from the nucleus into the cytoplasm and directs it to PD for cell-to-cell movement.

Apart from being methylated, P20 also undergoes phosphorylation at residue S11 in its ARM, which regulates the switch from a stable P20-harboring RNP complex for cell-to-cell movement to a P20-dissociated satBaMV RNA for translation in newly BaMV-coinfected cells (Vijayapalani et al. 2012). In this study, we demonstrate that P20 methylation functions in the formation of the satBaMV RNP complex and its autonomous long-distance trafficking. Crosstalk between phosphorylation and methylation has been reported previously for many proteins, such as FOX1 and CIRBP (Yamagata et al. 2008; Lenard et al. 2021). Therefore, dynamic changes in P20 methylation and phosphorylation catalyzed respectively by FIB MTase and other kinases, such as serine/threonine kinase-like protein and casein kinase 2α (Cheng et al. 2013; Hung et al. 2014), may direct the trafficking fate of satBaMV RNP complexes both intracellularly and intercellularly. How these protein modifications affect the long-distance movement of satBaMV in planta warrants further investigation.

## Materials and methods

### Plasmid construction

To generate pET15b-FIB, pET15b (Novagen) was digested with *Nde*I and *Xho*I, followed by ligation with the cDNA of *N. benthamiana* FIB, which had been amplified using FIB-*Nde*I-FP and FIB-*Xho*I-RP primers (Supplementary Table S1). To purify the FIB MTase single and triple mutants, we applied site-directed mutagenesis through fragment assembly (Agilent Technologies, Catalog #210518). The pET15b-FIB plasmid was used as the template for mutagenesis, and 3 polymerase chain reaction (PCR) primer pairs for R-to-A substitution were designed (FIB-K134A-F&R, FIB-D227A-F&R, and FIB-K256A-F&R) (Supplementary Table S1). To generate pET21b-P20 R5A, pET21b-P20 (Palani et al. 2009) was point-mutated by PCR using R5A-FP-F&R primers (Supplementary Table S1). To generate pBIN61-eGFP-NbFIB2, NbFIB2 with *Kpn*I and *Eco*RI sites was amplified by PCR using FIB-KpnI-F and FIB-EcoRI-R primers (Supplementary Table S1), before being fused to the pCass-N-eGFP plasmid. Then, the eGFP-NbFIB2 fragment was amplified by PCR with pCass-Tf-XmaI-F&R primers (Supplementary Table S1) and fused with pBIN61 at the *Xma*I site. To generate the NbFIB2 mutants for transient expression, pBIN61-eGFP-NbFIB2 plasmids were amplified using FIB-K134A-F&R, FIB-D227A-F&R, and FIB-K256A-F&R primers for site-directed mutagenesis. To generate pBIN61-P20 (R4A, R5A, R4/5A, or R4/5K)-eGFP, the full-length plasmids of the above-described mutants were amplified from the pBIN61-P20-eGFP template (Chang et al. 2016) by site-directed mutagenesis using primers R4A-F&R, R5A-F&R, R4/5A-F&R, and R4/5K-F&R (Supplementary Table S1). The same primers were used to create P20-mutated satBaMV (pKF4-P20R4A, pKF4-P20R5A, pKF4-P20R4/5A, and pKF4-P20R4/5K) from pKF4 (Lin et al. 1996; Liou et al. 2014) by site-directed mutagenesis. To generate pKF4GFP, P20-deficient satBaMV was purified from pBSGFP (Vijayapalani et al. 2012) using *Hin*dIII and *Bgl*II and then introduced into the pKn plasmid (Prasanth et al. 2011) with the same restriction enzyme sites. To generate pBIN61-P18-eGFP, pCass-P20-eGFP (Palani et al. 2006) was used as the template to create pCass-P18-eGFP by inverted PCR using P18-XmaI-F and pCass-invert-XmaI-R primers (Supplementary Table S1). Then, the P18-eGFP fragment was amplified from pCass-P18-eGFP by PCR with pCass-Tf-XmaI-F&R primers (Supplementary Table S1) and fused with pBIN61 at the *Xma*I site. To generate BIN61-N15-eGFP, N15 cDNA was amplified by PCR using N15-KpnI-F&R primers and fused with pCass-eGFP at the *Kpn*I site to create pCass-N15-eGFP. Then, N15-eGFP was amplified by pCass-Tf-XmaI-F&R primers and fused with pBIN61 at the *Xma*I site. The pFT-GFP and pGFP constructs from Dr. Tien-Shin Yu (Institute of Plant and Microbial Biology, Taipei, Taiwan) (Lu et al. 2012) were used to express FT or GFP RNA in plants. To generate pKF4-NLS or pKF4-NES, pCass-F4-NES and pCass-F4-NLS were generated by inverted PCR using pCBSF4 (Lin et al. 2004) as templates and the primers satBaMV-wt-R plus satBaMV-NES-F or satBaMV-NLS-F, respectively (Supplementary Table S1). The F4-NES/NLS fragment from pCass plasmids was cut and fused with pKn using *Hin*dIII and *Bgl*II to generate pKF4-NLS and pKF4-NES.

### Plant growth and grafting

All WT, Fib*i*, and satBaMV-transgenic *N. benthamiana* plants (Chang et al. 2016) were grown at 28 °C in a walk-in plant growth chamber under a 16 h light/8 h dark cycle with a light intensity of 185–222 μmol m^−2^ s^−1^ at the leaf surface. Forty-day-old *N. benthamiana* plants were used for cleft grafting (Turnbull et al. 2002), and each grafting experiment was repeated at least 3 times.

### Recombinant protein expression in *Escherichia coli*

To express the WT or MTase mutants of rFIB and rP20 proteins, the corresponding plasmids were transformed into *Escherichia coli* strain BL21. Recombinant proteins were harvested from *E. coli* by means of induction with 0.4 mm IPTG at 28 °C and purified through Ni^2+^ columns according to the manufacturer's instructions (GE Healthcare).

### Western blotting

Protein samples were separated on SDS–PAGE gels and transferred to PVDF membranes (Amersham Hybond P, GE10600023). The membranes were blocked with 5% nonfat milk, rinsed with PBST, and then incubated with primary and/or secondary antibodies. The rFIB was used for western blot analysis with anti-FIB antibody (Santa Cruz Biotech), followed by goat antirabbit IgG HRP secondary antibody (Abcam). Chemiluminescence images were taken after adding Clarity Western ECL substrate (Bio-Rad).

### In vitro and in vivo methylation

For in vitro FIB MTase assays, purified P20 protein was incubated with 4 μM rFIB WT or MTase mutants at 37 °C for 30 min in a reaction buffer (1 mm EDTA, 50 mm NaCl, 0.5 mm dithiothreitol, and 100 mm sodium phosphate pH 7.0) with ^3^H-SAM (PerkinElmer; 79 μCi/μmol) for scintillation counting. To identify the FIB-dependent methylation sites, 1 μg of the rP20, rP18, and N15 peptides was employed as substrates. After the reaction, 15 µL of the samples was fixed on a small Whatman filter. The filters were incubated in ice-cold 5% trichloroacetic acid (TCA) for 10 min and then washed twice with fresh 5% TCA for 10 min. Then, the filters were washed with ice-cold 100% ethanol for 5 min. After ethanol had been decanted, the dried filters were placed into scintillation vials (each containing 3 mL of scintillation liquid “Ultima Gold”; PerkinElmer), and the ^3^H-methylated products were quantified using a liquid scintillation counter (Beckman Coulter).

The following methylation-specific antibodies were used to detect the P20 methylated in vitro and in vivo by western blot analysis: anti-mme-R (anti-MMA, Abcam, [16B11], 1:500 dilution); anti-ASYM24 (anti-ADMA, Millipore, Cat#: 07-414, RRID: AB_310596, 1:1,000 dilution); anti-SYM10 (anti-SDMA, Millipore, Cat#: 07-412; RRID: AB_310594, 1:1,000 dilution); anti-Kme2 (anti-DMK, Invitrogen, Cat# PA5-96454; PA5-96454, 1:1,000 dilution); and anti-Qme (anti-MQ, Millipore, Cat#: ABS2185, Lot #Q2865523, 1:3,000 dilution). Monoclonal antitubulin antibody produced in mouse (Sigma-Aldrich, Cat#: T6074, RRID: AB_477582, 1:5,000 dilution) was used to detect tubulin levels as a loading control, and anti-histone H2 antibody (AS10 718, Agrisera) was obtained from Dr. Shu-Hsing Wu (Institute of Plant and Microbial Biology, Taipei, Taiwan).

For in vivo MTase assays, proteins from *N. benthamiana* coinfected with satBaMV or BaMV were immunoprecipitated by anti-P20 antibody and detected by western blotting (Palani et al. 2009; Chang et al. 2016).

### EMSA

The indicated amounts of purified rFIB or MTase mutants, along with P20 or its R5A mutant were incubated with 6 ng of ^32^P-labeled satBaMV riboprobe and 2 U of RNasin Ribonuclease Inhibitor (Promega) in 15 *µ*l of *N*-cyclohexyl-3-aminopropanesulfonic acid (CAPS) buffer for 20 min on ice. After incubation, samples were separated by 4∼12% NuPAGE (Catalog number: NP0321PK2, Thermo Fisher Scientific Inc.) with MES buffer (Catalog number: NP0002, Thermo Fisher Scientific Inc.) at 4 °C. After the gel had been dried and supported on 3MM Chr paper (Whatman), the mobility patterns of ^32^P-labeled nucleic acids were analyzed using a Phosphor-Imager with ImageQuant Version 3.3 (Molecular Dynamics). Calculations of rFIB and rP20 enzyme kinetics were conducted according to a previous report (Vijayapalani et al. 2012).

### RNA analysis

Total RNA was extracted from *N. benthamiana* tissues using TriPure following the manufacturer's instructions (Roche, Switzerland). Northern blot analysis was carried out as described previously (Lin et al. 1996). SatBaMV accumulation was analyzed using ^32^P-labeled RNA probes specific for the full-length satBaMV generated from *EcoR*I-linearized pBSHE (Lin et al. 1996). FT and GFP RNA accumulations were analyzed as previously described (Lu et al. 2012). To analyze satBaMV, FT or GFP RNA by RT-PCR, respective cDNAs synthesis and detection were performed using SuperScript III RT-PCR system (Invitrogen) according to the manufacturer's instructions, followed by PCR as previously described (Chang et al. 2016).

### RIP

RIP with anti-P20 antibody was performed as described by Chang et al. (Chang et al. 2016). Briefly, crude protein extracts were prepared in extraction buffer (50 mm Tris-Cl (pH 7.2), 100 mm NaCl, 10% glycerol, 1 mm phenylmethylsulfonyl fluoride (PMSF), 10 μM MG132, and cOmplete protease inhibitors (Roche)) (Dharmasiri et al. 2005). Approximately 1 mg of protein extract was incubated with the anti-P20 antibody (diluted 1:150 v/v) at 4 °C for 1 h, followed by an additional incubation with 20 μL of nProtein A Sepharose 4 Fast Flow (GE Healthcare) for 3 h on a rotary shaker. The beads were then washed 3 times with washing buffer (extraction buffer without MG132), resuspended in washing buffer, and divided into 2 portions for immunoblotting and RNA extraction.

### Tissue blotting

Sections were cut from fresh stem tissues by hand using a new razor blade. Tissue blots were made by pressing the newly cut surface onto a membrane (Amersham Hybond-N, GE Healthcare) and detected using a ^32^P-labeled RNA probe (Lin et al. 2013).

### Mass analysis

The procedure for analyzing methylated N15 mixtures using a MALDI-TOF/TOF-MS/MS instrument based on the footprint of a new ultrafleXtreme spectrometer (Bruker) was previously described (Pham et al. 2015). The sensitivity for interpretable collision-induced dissociation (CID) spectra of peptide standards is in the range of 1–10 fmol deposited on the target. New modifications were defined in MaxQuant, with the mass increment and residue specificities corresponding to the heavy versions of methylated arginine. The peptide pairs were separated into 2 classes based on the following criteria: peptides were assigned to Class A with a score >45; for Class B, the peptides were identified by a score <45; with the presence of a hmSILAC pair being used to assign confidence to the identification. To ensure that arginine modification positions were accurately assigned, a localization probability threshold of >0.95 was applied.

### BaMV ORF1-HA transgenic *N*. *benthamiana*

To generate BaMV ORF1-HA transgenic plants, the pEpyon-based binary vector containing BaMV ORF1 tagged with HA (Lee et al. 2011) was used for *Agrobacterium*-mediated transformation in *N*. *benthamiana* according to a previous report (Lin et al. 2013). Line 15-2-1 of ORF1-HA transgenic *N*. *benthamiana* was used in all experiments.

### *Agrobacterium* culture, satBaMV inoculation and transient expression

To express WT or MTase mutants of FIB in *N. benthamiana* leaves, *Agrobacterium* C58C1 harboring the relevant plasmids was incubated in inoculation buffer (10 mm MES, 10 mm MgCl_2_, 200 μM acetosyringone) and diluted to an optical density (OD) of 1.0 at 600 nm for infiltration into plant leaves as described previously (Liou et al. 2014).

For BaMV and satBaMV transient expression, *Agrobacterium* C58C1 containing pKB and pKF4 were grown and coinfiltrated into leaves of *N. benthamiana* plants as described previously (Chang et al. 2016).

To visualize the subcellular localization of P20 mutants and FIB in leaf epidermal cells, eGFP-tagged P20 (pBIN61-P20-eGFP), its mutants (N15, P18, R4A, R5A, R4/5A, R4/5 K), pBIN61-eGFP-NbFIB2, or pBIN61-mCherry-NbFIB2 (Chang et al. 2016) in *Agrobacterium* C58C1 was used for agroinfiltration. We used DAPI (Roche) and aniline blue fluorochrome (Biosupplies Australia) as DNA and callose markers, respectively (Chang et al. 2016).

To investigate the PD targeting of FIB, the leaves of ∼3-week-old BaMV ORF1-HA transgenic *N. benthamiana* were first inoculated with satBaMV F4 or F5 transcript according to a previous report (Lin et al. 1996). After 5 days, *Agrobacterium* C58C1 containing pBIN61-eGFP-NbFIB2 was infiltrated, and then 2 days later the leaves were analyzed by confocal microscopy.

To analyze the impact of P20 mutants on long-distance satBaMV transport, *Agrobacterium* C58C1 carrying pKF4, pKF4-P20R4A, pKF4-P20R5A, pKF4-P20R4/5A, pKF4-P20R4/5 K, pKF4GFP or pKF5 was cultured as previously described and infiltrated into 6-week-old WT *N. benthamiana* grafted plants as previously described (Chang et al. 2016). *Agrobacterium* C58C1 containing pFT-GFP or pGFP was used as a control.

To analyze the effects of P20 nuclear localization on long-distance satBaMV transport, *Agrobacterium* C58C1 carrying pKF4, pKF4-NLS or pKF4-NES was agroinfiltrated into WT *N. benthamiana* grafted plants as described previously (Chang et al. 2016).

### Cell fractionation

Nuclei from *N. benthamiana* were isolated according to a procedure described previously (Kinkema et al. 2000) with slight modification. In brief, tissues were homogenized in Honda buffer (2.5% Ficoll 400, 5% dextran T40, 0.4 m sucrose, 25 mm Tris-HCl pH 7.4, 10 mm MgCl_2_, 10 mm β-mercaptoethanol, and a proteinase inhibitor cocktail) by using a mortar and pestle, and then filtered through a 62-μm (pore-size) nylon mesh. Triton X-100 was added to a final concentration of 0.5%, and the mixture was incubated on ice for 15 min. The solution was centrifuged at 1500 × *g* for 5 min, and the pellet was washed with Honda buffer containing 0.1% Triton X-100. The pellet was resuspended gently in 1 mL of Honda buffer and transferred to a microcentrifuge tube. This nucleus-enriched preparation was first centrifuged at 100 × *g* for 1 min to pellet the starch and cell debris, followed by centrifugation at 1800 × *g* for 5 min to pellet the nuclei.

The membrane fraction was isolated according to a procedure described previously (LaMontagne et al. 2016) with slight modification. In brief, the nuclei-depleted fraction as described above was ultracentrifuged for 30 min at 100,000 × *g* and 4 °C. The pellet was completely resuspended in homogenization buffer (50 mm HEPES-KOH, pH 7.5, 250 mm sucrose, 5% glycerol, 10 mm EDTA, 0.5% PVP-10, 50 mm NaPP, 1 mm NaMo, 25 mm NaF, 3 mm DTT, 1 mm PMSF, 10 μM leupeptin, 10 nm calyculin). The samples were solubilized using 1% detergent in homogenization buffer and incubated on ice for 30 min. After incubation, the samples were ultracentrifuged for 30 min at 100,000 × *g* and 4 °C, and the supernatant was carefully collected for SDS–PAGE followed by western blot analysis.

### Confocal imaging

*N. benthamiana* plants were used for agroinfiltration to observe the subcellular localizations of eGFP or mCherry fusion proteins. Confocal microscopy was carried out 2 days after agroinfiltration. Images were captured using a Leica STELLARIS 8 confocal microscope (Leica Microsystems) or a Zeiss LSM880 Airyscan confocal microscope (Zeiss) with Airyscan mode (super-resolution). The eGFP, mCherry, aniline blue, and DAPI were excited with wavelengths at 488, 543, 405, and 405 nm, respectively. The detection wavelengths were optimized according to fluorophore groups in each experiment to avoid emission bleeding. For Leica confocal microscopy, the TauGating threshold for eGFP was set between 1.034 and 8 ns to eliminate chloroplast reflection. The PCC and the distribution of fluorescence were calculated using the ZEN 3.7 blue software of the LSM880 confocal microscope.

### Statistical analysis

Quantitative data were analyzed using Student's *t*-test or ANOVA, followed by Tukey's test to determine statistical significance (Supplementary Data Set 1). Data are presented as mean ± SD from at least 3 independent biological replicates, each comprising a minimum of 3 plants or samples per treatment.

### Accession numbers

Sequence data from this article can be found at NCBI GenBank (https://www.ncbi.nlm.nih.gov/) under accession numbers NbFIB2 (CAK32531.1), P20 (NP_612559.1), and satBaMV (L22762.1).

## Supplementary Material

koaf224_Supplementary_Data

## Data Availability

All data supporting the findings of this study are available within the article and its supplementary materials.

## Supplementary data

The following materials are available in the online version of this article.

**Supplementary Figure S1.** Alignment of FIB amino acid sequences.

**Supplementary Figure S2.** Long-distance movement of BaMV in Fib*i* scions grafted onto *N*. *benthamiana* plants.

**Supplementary Figure S3.** MS/MS analysis of methylation in the N15 ARM region of P20.

**Supplementary Figure S4.** Distribution of P20-eGFP or mutants colocalized with PD.

**Supplementary Figure S5.** Subcellular localizations of FIB with P20 or the R5A mutant in *N*. *benthamiana* cells.

**Supplementary Figure S6.** SatBaMV-triggered relocalization of FIB.

**Supplementary Figure S7.** Nuclear localization of WT P20 and mutant variants.

**Supplementary Figure S8.** Protein–protein interactions between P20 and importin α1 or α2 in yeast 2-hybrid assay.

**Supplementary Table S1.** Primer sequences used in this study.

**Supplementary Data Set S1.** Results of statistical analyses.

## Dive Curated Terms

The following phenotypic, genotypic, and functional terms are of significance to the work described in this paper:

PRMT6 Gramene: AT3G20020
PRMT6 Araport: AT3G20020

BSMV Gramene: Barley stripe mosaic virus
BSMV Araport: Barley stripe mosaic virus

NCBI Gramene: https://www.ncbi.nlm.nih.gov
NCBI Araport: https://www.ncbi.nlm.nih.gov
