# Supplementary material for: Nucleolar fibrillarin methyltransferase regulates systemic trafficking of a plant virus satellite RNA
Source: Plant Cell. 2025 Sep 22;37(10):koaf224. doi: 10.1093/plcell/koaf224 (PMC12510318; doi:10.1093/plcell/koaf224)
Supplement: koaf224_Supplementary_Data [file koaf224_supplementary_data.zip › Supplementary Data.pdf]

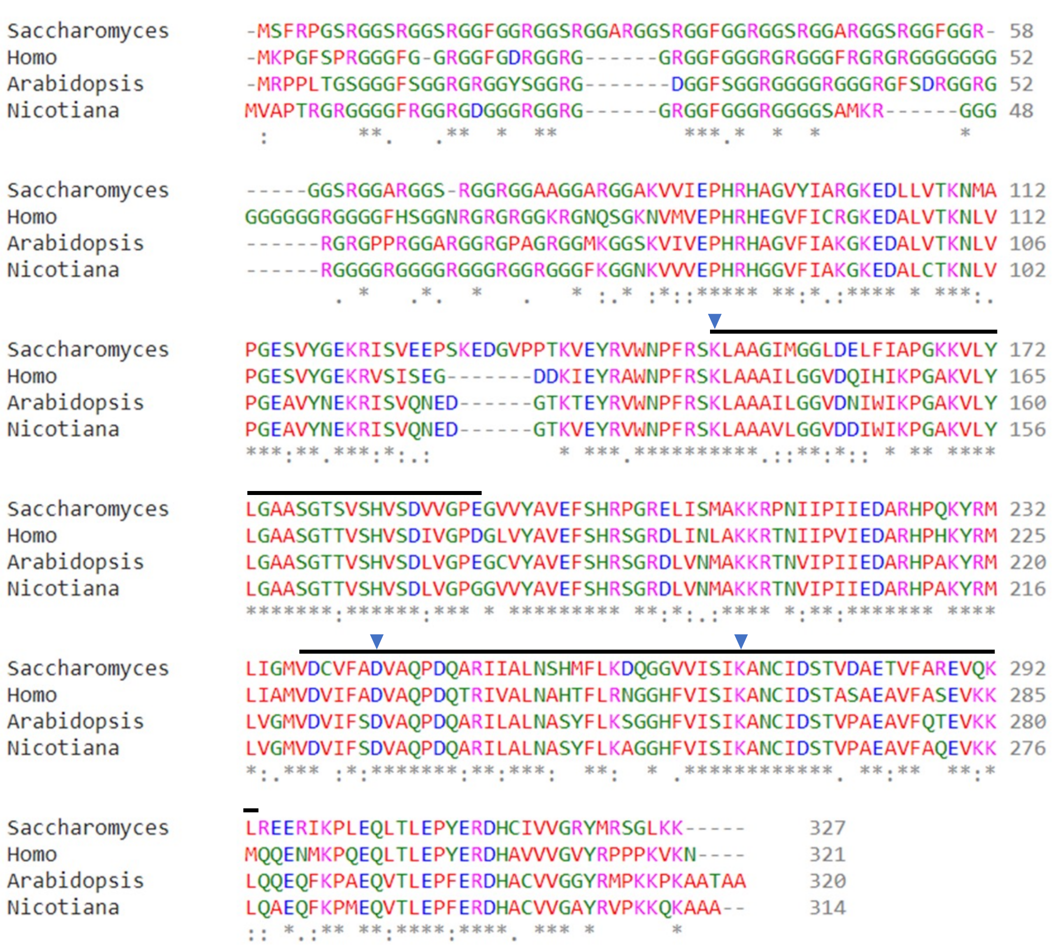

Supplementary Figure S1. Alignment of FIB amino acid sequences. (Supports Figure 1). This figure shows the alignment of FIB amino acid sequences across different species. Conserved residues are marked with stars. The black lines highlight the RNA-binding site of AtFib2, while the arrowheads indicate the catalytic triad of NbFib2. Species included in the alignment are Saccharomyces (*Saccharomyces cerevisiae*); Homo (*Homo sapiens*); Arabidopsis (*Arabidopsis thaliana*); Nicotiana (*Nicotiana benthamiana*).

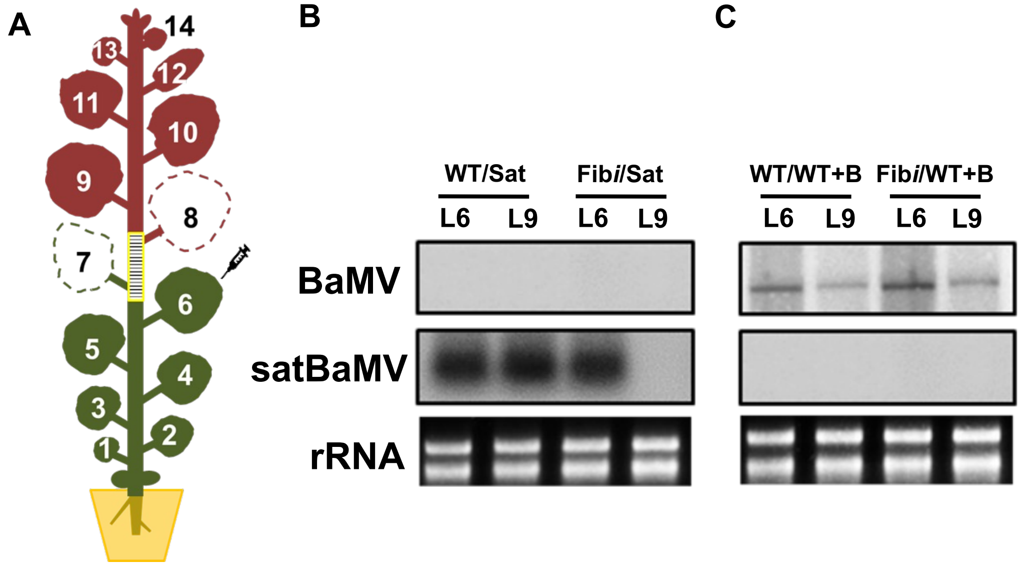

Supplementary Figure S2. Long-distance movement of BaMV in Fib*i* scions grafted onto *N. benthamiana* plants. **(Supports Figure 2). A,** Illustration of the grafting experiment on 40-day-old *N. benthamiana* plants. **B-C,** WT or Fib*i* scions were grafted onto satBaMV transgenic plants (Sat) (B), or the WT stock agroinfiltrated with a plasmid carrying BaMV-eGFP (+B) at L6 leaves (C). The northern blotting of BaMV-eGFP and satBaMV accumulation in the stock (L6) and scion (L9) leaves at 9 DPA, using specific probes. Results are representative of three independent experiments.

A

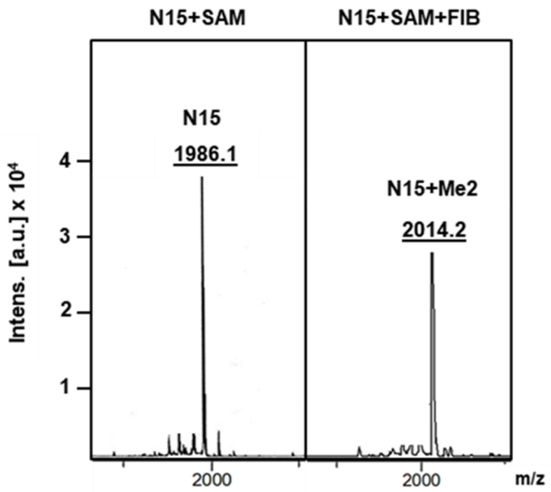

B

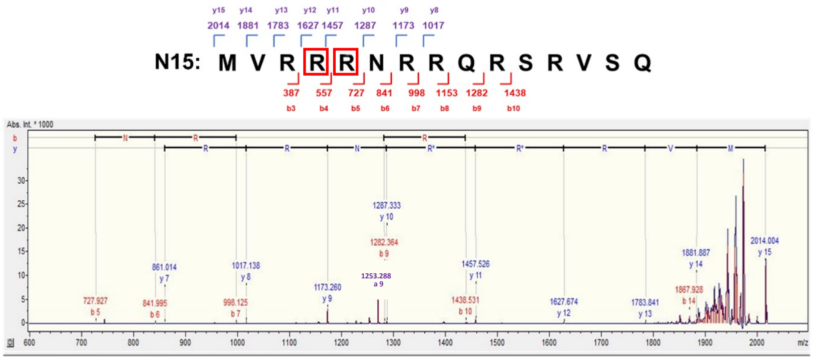

Supplementary Figure S3. MS/MS analysis of methylation in the N15 ARM region of P20. (Supports Figure 3). A, Mass spectrometry (MS) analysis of N15 methylation by rFIB. Methylation of the N15 region was assessed in the presence (right) or absence (left) of rFIB treatment with 1 mM SAM using MALDI-TOF MS. **B,** Tandem mass spectrometry (MS/MS) analysis of methylated N15 by rFIB. Representative MS/MS spectra of the N15 ions from FIB-mediated methylation, identified via MALDI-TOF/TOF MS. The inset provides a schematic summary of the observed fragment ions for N15. The series of b or y ions, spanning from b3 to b10 and y8 to y15, indicate monomethylation at residues R4 and R5 (highlighted in red columns). MS/MS spectra were obtained from three independent biological replicates, each with at least two technical replicates per sample.

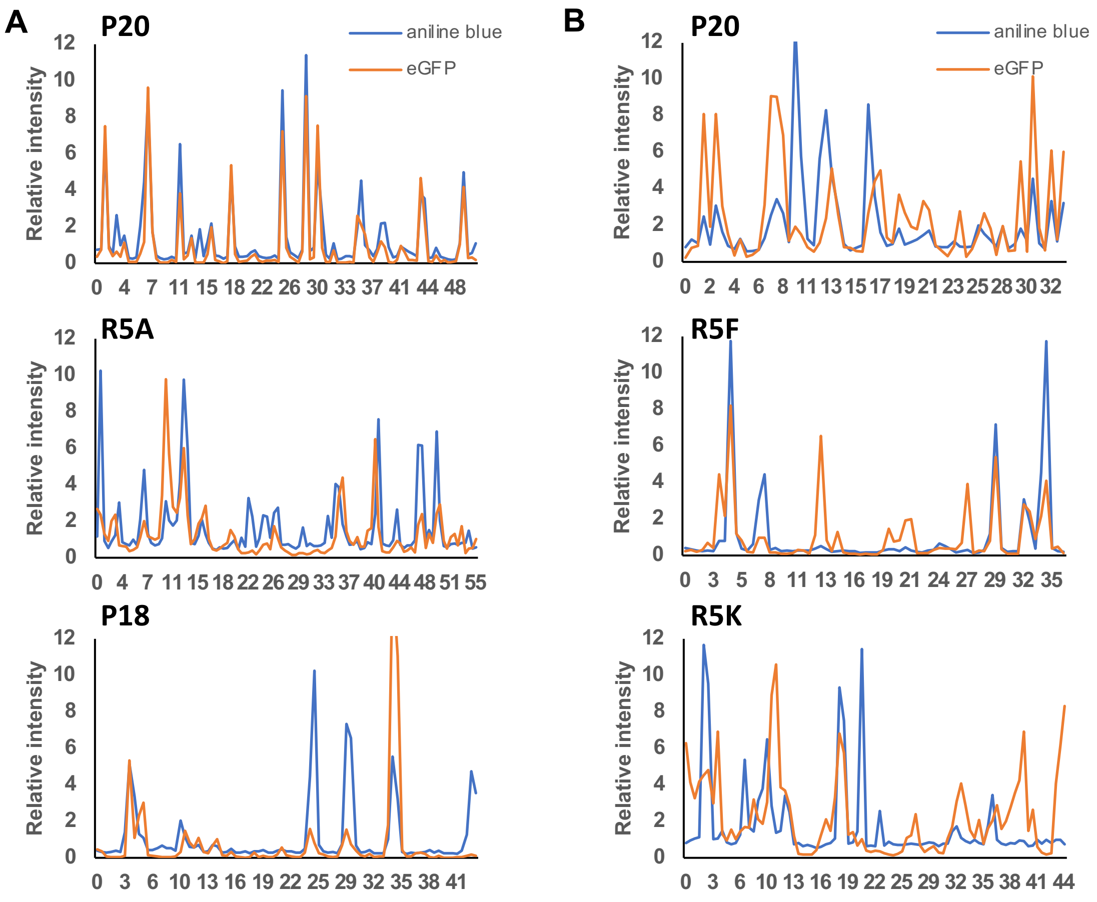

Supplementary Figure S4. Distribution of P20-eGFP and mutants colocalized with PD. (Supports Figure 5). *N. benthamiana* leaves were infiltrated with *Agrobacterium* carrying constructs for P20-eGFP, P20 R5A-eGFP, P18-eGFP (A) or P20 R5F-eGFP, P20 R5K-eGFP (B). At 2 DPA, leaf sections were stained with aniline blue to mark PD followed by confocal microscopy. Relative intensities of P20-eGFP or its mutants, alongside aniline blue, along the cell wall were quantified using ZEN 3.7 blue software. Overlay peaks with relative intensity values greater than 2 were identified as the localization of P20 proteins at PD. The X-axis represents the distance (um).

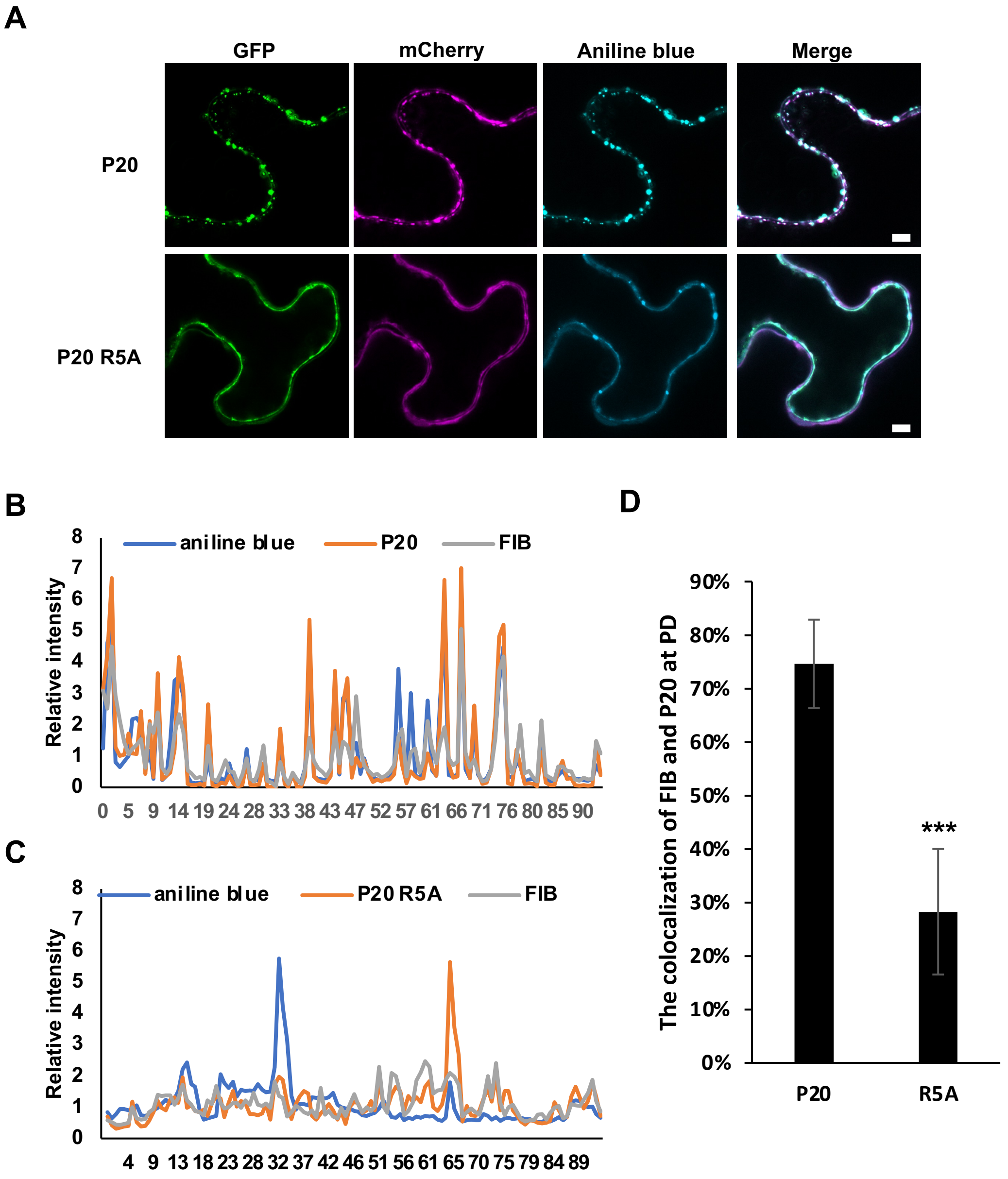

Supplementary Figure S5. Subcellular localizations of FIB with P20 or the R5A mutant in *N. benthamiana* cells. **(Supports Figure 5). A,** Confocal images showing the localization of P20 and the R5A mutant with aniline blue at PD in *N. benthamiana.* Leaves were agroinfiltrated with plasmid carrying mCherry-NbFIB2 and P20-eGFP or P20 R5A-eGFP, respectively, stained with aniline blue and imaged at 2 DPA using a Zeiss LSM880 Airyscan Confocal Microscope. Scale bars: 5 µm. **B-C,** Relative fluorescent intensities of FIB and aniline blue colocalized with P20 **(B)** or P20 R5A **(C)** along the cell periphery shown in **A**. Signal distributions were analyzed using ZEN 3.7 Blue software. The X-axis represents the distance (µm). **D,** The percentage of colocalization of FIB and P20 proteins at PD, marked by aniline blue. Signal distributions of FIB, P20, and aniline blue were quantified using ZEN 3.7 Blue software. Colocalization at PD was determined by the overlap of the three intensity peaks shown in **B** and **C**. Data, represented as mean ± SD, were analyzed using Student's t-test (*** $P<0.001$; n=9).

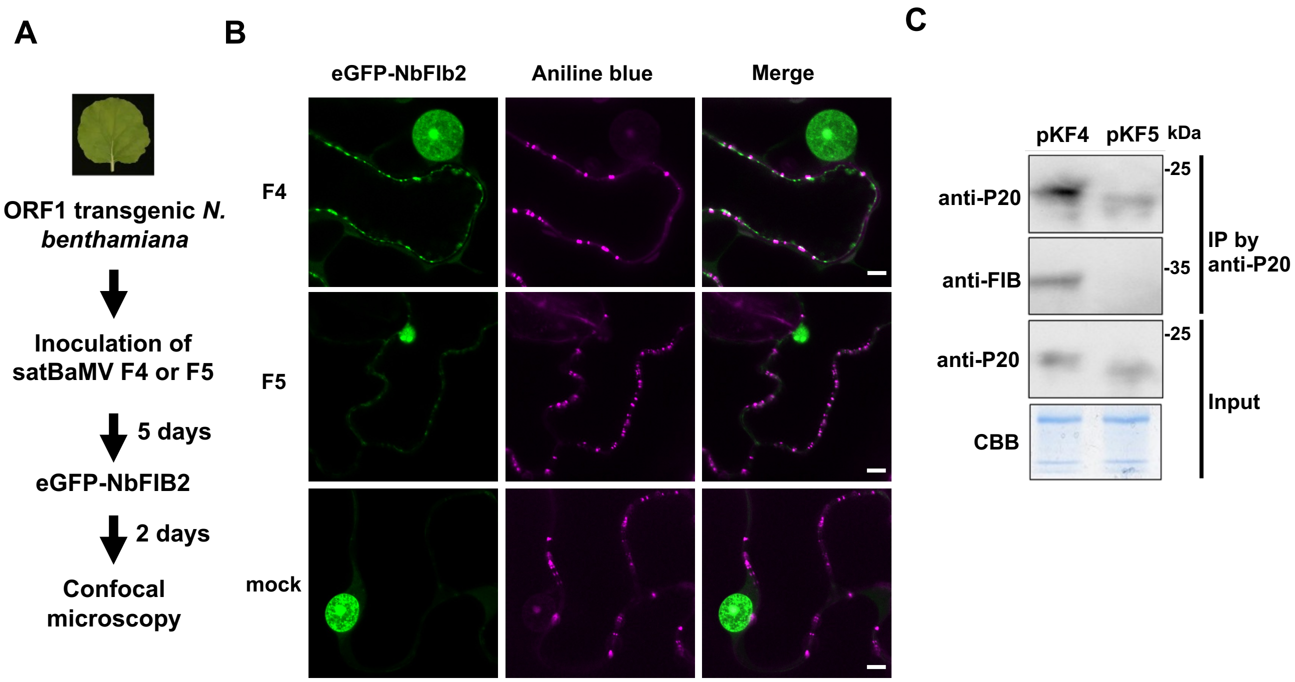

Supplementary Figure S6. SatBaMV-triggered relocalization of FIB. (Supports Figure 6). A, Flowchart illustrating the procedure used to visualize the subcellular localization of eGFP-NbFIB2 after inoculation with satBaMV F4 or F5. Image of ORF1 transgenic *N. benthamiana* was reused from Figure 6A due to identical treatment and conditions. **B,** Subcellular localization of eGFP-NbFIB2 following inoculation with satBaMV F4 or F5. As shown in panel **A**, leaf sections were stained with aniline blue and observed using a Zeiss LSM880 Airyscan Confocal Microscope. Scale bars: 5 µm. **C,** Interaction of P20 or P18 with FIB. Total proteins were extracted from WT *N. benthamiana* leaves agroinfiltrated with pKF4 or pKF5 at 7 DPA and subjected to co-IP using anti-P20 antibody. Western blotting was conducted with anti-P20 and anti-FIB antibodies, with CBB staining serving as a loading control. The results presented are representative of three independent co-IP experiments with similar outcomes.

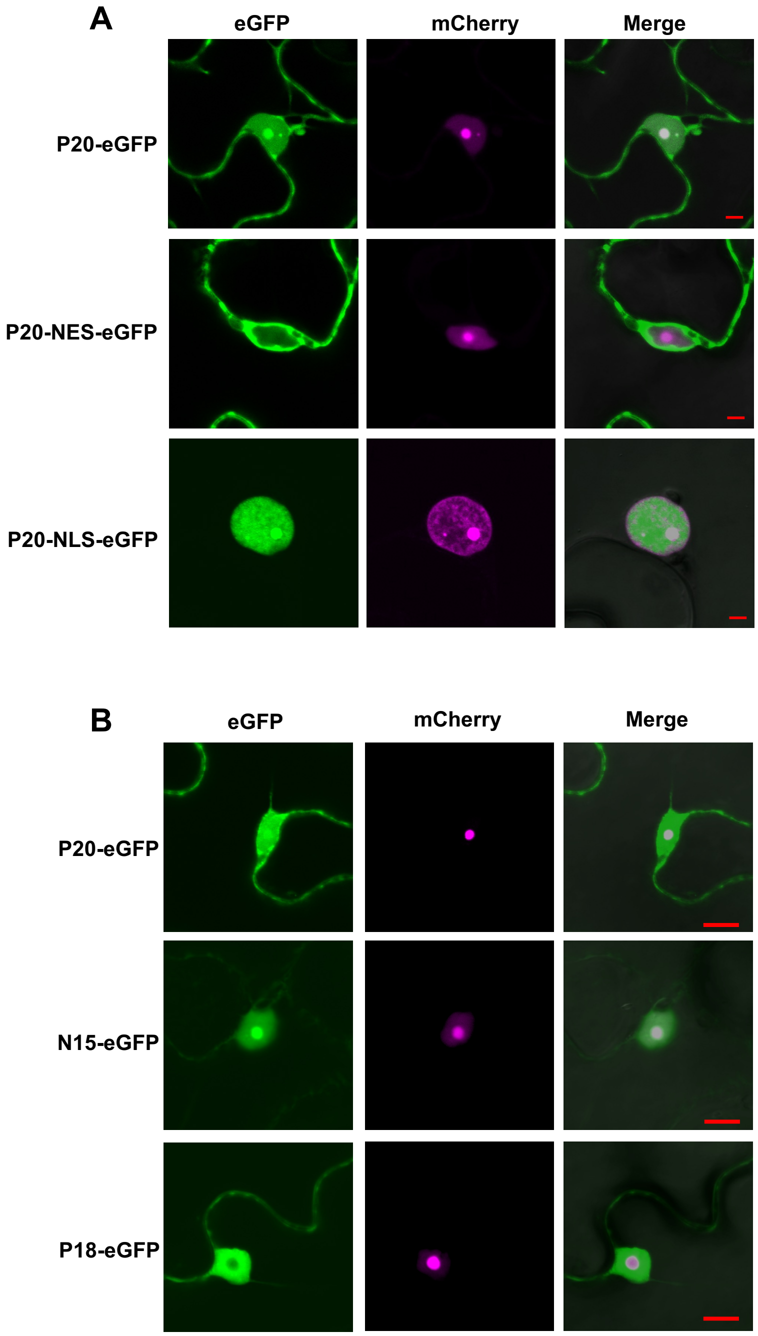

Supplementary Figure S7. Nuclear localization of WT P20 and mutant variants. (Supports Figure 7). Leaves of *N. benthamiana* were agroinfiltrated with plasmids expressing P20-eFGP and its mutant variants, and confocal microscopy was performed at 2 DPA. mCherry-NbFIB2 was used as a nucleolar marker. **A**, Localizations of P20-eGFP, P20-NES-eGFP, and P20-NLS-eGFP. Scale bars: 5 µm. **B**, Localizations of P20-eGFP, N15-eGFP, and P18-eGFP. Scale bars: 10 µm.

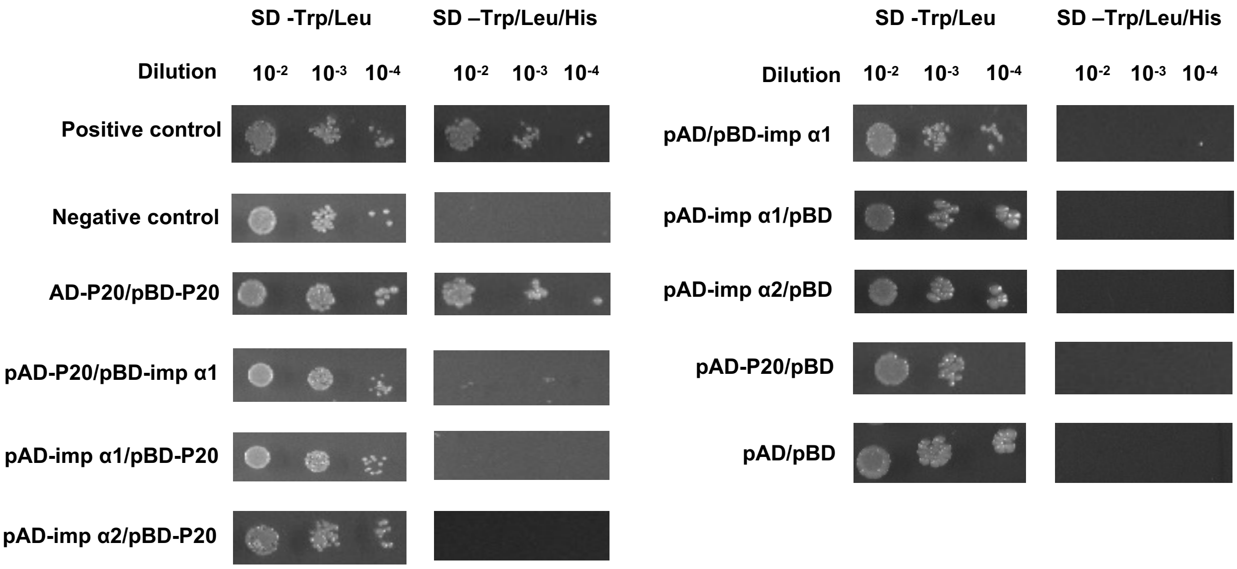

Supplementary Figure S8. Protein-protein interactions between P20 and importin α1 or α2 in yeast two-hybrid assay. (Supports Figure 7). Yeast cells were transformed with the indicated constructs and grown on synthetic dropout media (SD) lacking Trp/Leu/His or Trp/Leu to assess interactions. The positive control used was pAD-SV40/pBD-P53, while the negative control was pAD-SV40/pBD-lamin C.

Supplementary Table S1. Primer sequences used in this study. This table provides a comprehensive list of the primer sequences utilized throughout the study.

Name	Enzyme	Sequence (5'→3') [a]
**FIB-NdeI-F**	NdeI	GGAATTCCATATGGCAGCCATGGTTGCACCAAC
**FIB-XhoI-R**	XhoI	CCCTCGAGGCTTAAATTTTCTAGGCAGC
**FIB-KpnI-F**	KpnI	AAAGGTACCATGGTTGCACCAACTAGAGG
**FIB-EcoRI-R**	EcoRI	AAAGAATTCCTAGGCAGCAGCCTTCTGCTT
**FIB-K134A-F**		ATCCCTTCCGTTCTGCGTTAGCAGCTGCA
**FIB-K134A-R**		TGCAGCTGCTAACGCAGAACGGAAGGGAT
**FIB-D227A-F**		GATGTGATATTTTCTGCGGTTGCTCAG
**FIB-D227A-R**		CTGAGCAACCGCAGAAAATATCACATC
**FIB-K256A-F**		TGTTATGTCAATCGCGGCCAACTGCATAGAT
**FIB-K256A-R**		ATCTATGCAGTTGGCCGCGATTGACATAACA
**R4A-F**		ATGGTTCGGGCAAGAAATCGTCGCC
**R4A-R**		GGCGACGATTTCTTGCCCGAACCAT
**R5A-F**		ATGGTTCGGAGGGCAAATCGTCGCC
**R5A-R**		GGCGACGATTTGCCCTCCGAACCAT
**R4/5A-F**		ATGGTTCGGGCAGCAAATCGTCGCC
**R4/5A-R**		GGCGACGATTTGCTGCCCGAACCAT
**R4/5K-F**		ATGGTTCGGAAGAAGAATCGTCGCC
**R4/5K-R**		GGCGACGATTCTTCTTCCGAACCAT
**satBaMV-NES-F**		AACGAGCTTGCCTTGAAATTAGCAGGGTTAGACATAAACAAA<br>TGATCCACGAGCACAACC
**satBaMV-NLS-F**		AAGAGGCGCCGCAGGCGATGATCCACGAGCACAACC
**satBaMV-wt-R**		ACTGGTTGGCGCACGGTCAG
**P18-XmaI-F**	XmaI	ATCGCCCGGGATGACCGACATCATGTATGGCTC
**pCass-invert-XmaI-R**	XmaI	ATCGCCCGGGTTTTAGGCCTCTCCAAATGAAATG
**pCass-Tf-XmaI-F**	XmaI	ATCGCCCGGGGGAAGTTCATTTCATTTGGAGAGG
**pCass-Tf-XmaI-R**	XmaI	ATCGCCCGGGGGTGATTTCAGCGTACCGAATTC
**N15-KpnI-F**	KpnI	AAGGTACCATGGTTCGGAGGAGAAATCGTC
**N15-KpnI-R**	KpnI	AAGGTACCTTA TTG GGA GAC ACG CGA TCT CT

[a] Restriction enzyme sites are underlined.
